# A photoactivatable crosslinking system reveals protein interactions in the Toxoplasma gondii inner membrane complex

**DOI:** 10.1371/journal.pbio.3000475

**Published:** 2019-10-04

**Authors:** Charles Paul Choi, Andy Seong Moon, Peter Sungmin Back, Yasaman Jami‐Alahmadi, Ajay Amar Vashisht, James Akira Wohlschlegel, Peter John Bradley

**Affiliations:** 1 Molecular Biology Institute, University of California, Los Angeles, Los Angeles, California, United States of America; 2 Department of Microbiology, Immunology and Molecular Genetics, University of California, Los Angeles, Los Angeles, California, United States of America; 3 Department of Biological Chemistry, David Geffen School of Medicine, University of California, Los Angeles, Los Angeles, California, United States of America; Johns Hopkins University Bloomberg School of Public Health, UNITED STATES

## Abstract

The *Toxoplasma gondii* inner membrane complex (IMC) is an important organelle involved in parasite motility and replication. The IMC resides beneath the parasite’s plasma membrane and is composed of both membrane and cytoskeletal components. Although the protein composition of the IMC is becoming better understood, the protein–protein associations that enable proper functioning of the organelle remain largely unknown. Determining protein interactions in the IMC cytoskeletal network is particularly challenging, as disrupting the cytoskeleton requires conditions that disrupt protein complexes. To circumvent this problem, we demonstrate the application of a photoreactive unnatural amino acid (UAA) crosslinking system to capture protein interactions in the native intracellular environment. In addition to identifying binding partners, the UAA approach maps the binding interface of the bait protein used for crosslinking, providing structural information of the interacting proteins. We apply this technology to the essential IMC protein ILP1 and demonstrate that distinct regions of its C-terminal coiled-coil domain crosslink to the alveolins IMC3 and IMC6, as well as IMC27. We also show that the IMC3 C-terminal domain and the IMC6 N-terminal domain are necessary for binding to ILP1, further mapping interactions between ILP1 and the cytoskeleton. Together, this study develops a new approach to study protein–protein interactions in *Toxoplasma* and provides the first insight into the architecture of the cytoskeletal network of the apicomplexan IMC.

## Abstract 2

The inner membrane complex of the human parasite Toxoplasma gondii is an important organelle involved in motility and replication. This study expands the genetic code of Toxoplasma, allowing the use of photoactivatable unnatural amino acids to uncover interactions within the apicomplexan inner membrane complex.

## Introduction

The phylum Apicomplexa consists of some of the most successful eukaryotic intracellular parasites in the world. Apicomplexans that cause disease in humans include *T*. *gondii*, which causes toxoplasmosis in immunocompromised patients and congenitally infected neonates, *Plasmodium* spp., which cause malaria, and *Cryptosporidium* spp., which are major causes of diarrheal disease in children [1–3]. Other members of the phylum such as *Eimeria*, *Theileria*, *Babesia*, and *Neospora* are veterinary pathogens and result in billions of dollars in losses per year worldwide in the poultry and cattle industries [4–6]. *T*. *gondii* serves as a model organism for the study of apicomplexan biology due to its relative ease of continuous culture, high rate of transformation, and a robust set of tools for genetic manipulation and functional analyses.

Apicomplexans exhibit a number of specialized organelles that enable them to occupy their intracellular niche. One of these is the inner membrane complex (IMC), a unique structure that underlies the plasma membrane of the parasite and consists of flattened membrane vesicles supported by a cytoskeletal filament network [7]. The membrane and cytoskeletal components of the IMC work in concert to perform critical roles in the lytic cycle of the parasite. First, the IMC houses the glideosome, the actomyosin motor complex that enables gliding motility and host cell invasion [8]. Second, it serves as the scaffold for the apicomplexan replication process of internal budding, in which daughter cells are formed within the maternal cytoplasm, ultimately adopting the maternal plasma membrane and yielding progeny [9]. The asexual stages of *Toxoplasma* undergo endodyogeny, in which two daughter cells are produced per maternal parasite with each replication cycle. Other apicomplexans often replicate using variations of this internal budding process called schizogony or endopolygeny, in which multiple rounds of nuclear replication and karyokinesis result in the generation of up to 64 daughters at once [10].

In *Toxoplasma*, the IMC is partitioned into a cone-shaped apical cap (a coccidian-specific structure), a central body portion characterized by an array of rectangular membrane plates, and a basal complex, which is responsible for closure of the daughter buds to complete division [11,12]. Interestingly, recent studies using in vivo proximity-dependent biotin labeling (BioID) and other approaches have revealed that most IMC proteins localize to only one of these subregions [13,14]. Similarly, detergent solubilization studies have shown that the apical cap and body contain separate groups of membrane-associated and cytoskeleton-associated proteins, supporting the idea that each section is composed of specialized cargo that serves varying purposes. While the membrane and cytoskeletal layers are distinct, they are closely associated with each other through protein–protein interactions and fatty acylations that can tether cytoskeletal proteins to the membrane vesicles of the organelle.

Despite an increased understanding of the protein constituents that make up the IMC, the precise roles of most of these proteins and how they are organized remain largely unknown. The filamentous network of the IMC is believed to be formed by the alveolins, a family of 14 proteins that are characterized by a poorly conserved proline and valine-rich alveolin repeat domain [15,16]. The alveolins have different localizations within the three IMC subregions and likely serve roles in providing structural support for each of these compartments. However, the identification of many non-alveolin detergent-insoluble IMC proteins suggests that the IMC cytoskeleton is a complex structure whose organization remains enigmatic. One such protein is IMC localizing protein 1 (ILP1), which is an IMC body protein that is enriched in forming daughter buds, similar to a subgroup of alveolins (IMC3/6/10) [17]. We have demonstrated that conditional knockout of ILP1 causes a collapse of IMC integrity and inability to properly replicate, a lethal phenotype for the parasite [13]. Interestingly, the *Plasmodium* ortholog of ILP1 (*Pf*G2) is not essential, but its disruption results in significant morphological changes in ookinetes and sporozoites, reduced motility, and a loss of sporozoite infectivity [18]. How ILP1 imparts structural stability to the parasite would be best understood by determining its binding partners, but studying protein–protein interactions in the IMC cytoskeleton is typically challenging due to its detergent-insoluble nature.

One approach to overcome this difficulty is the use of inducible crosslinking unnatural amino acids (UAA). The UAA technology involves the expansion of the genetic code using an orthogonal aminoacyl-tRNA synthetase and amber stop codon (TAG) suppressor tRNA pair [19,20]. The tRNA is charged with the desired synthetic UAA and can be used by the endogenous ribosomal machinery to incorporate the UAA into the primary sequence of a protein of interest at an engineered in-frame amber stop codon. The UAA *p*-azidophenylalanine (*Azi*) belongs to a class of photoreactive crosslinkers called aryl azides and forms a highly reactive nitrene moiety upon exposure to relatively nondestructive 365-nm (UV-A) light [21,22]. Because Azi is a zero-length crosslinker, it should only form crosslinks when the UAA is positioned within the binding interface of the bait protein and its partner. Successful crosslinking of the bait can be observed as a higher molecular weight species by western blot. The bound partner can then be verified by immunoblot when the crosslinked partner is suspected, or purified and identified by mass spectrometry if unknown.

Here, we report the successful implementation of a photoactivated UAA crosslinking system in *T*. *gondii* and the application towards uncovering protein interactions within the intricate multiprotein IMC cytoskeleton complex. We first demonstrate that this system functions efficiently in the parasite in terms of both Azi incorporation and photocrosslinking. We then use this technology to show that ILP1 directly interacts with multiple components of the cytoskeleton of the IMC, revealing the first organization of this organellar compartment and identifying the precise binding regions through which ILP1 associates with the IMC network. This photoactivatable UAA system provides a unique tool to dissect additional protein–protein interactions that will help to unravel aspects of *T*. *gondii* cell biology that may have historically been difficult to study.

## Results

### Adaptation of the photoreactive UAA crosslinking system in *T*. *gondii*

**Fig 1:**
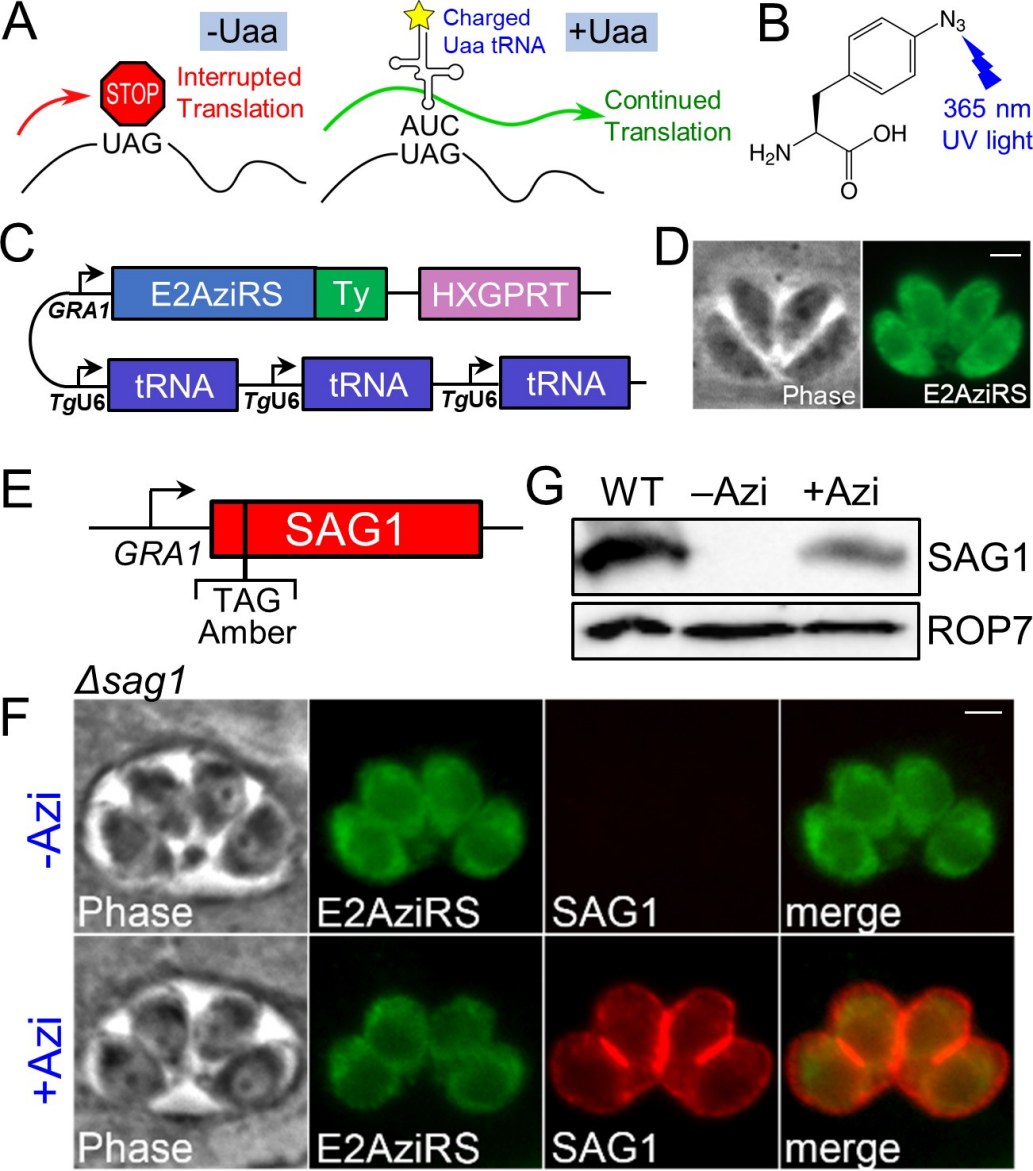
Engineering the UAA system and demonstrating efficient incorporation of Azi in *T*. *gondii*. (A) Diagram showing the use of amber stop codon suppression to incorporate UAAs into a nascent peptide strand using the endogenous translation machinery. (B) Chemical structure of the photoreactive UAA *p*-azidophenylalanine (Azi). Exposure of Azi to UV-A (365-nm) ultraviolet light causes the azide group to irreversibly form a reactive nitrene intermediate, which forms covalent crosslinks with proximal proteins. (C) Construct showing the Ty1-tagged aminoacyl-tRNA synthetase (E2AziRS) driven by the constitutively active *GRA1* promoter and three tandem cassettes of the cognate amber suppressor tRNA driven by the RNA polymerase III–specific U6 promoter. (D) IFA showing that stably expressed E2AziRS localizes to the parasite cytoplasm as expected. Green, mouse anti-Ty1. Scale bar represents 2 μm. (E) Construct showing the *SAG1* gene driven by the *GRA1* promoter, in which the second amino acid F2 has been mutated to an amber codon. (F) IFA showing RH*ΔhxgprtΔsag1* parasites stably transfected with the synthetase/tRNA and SAG1 constructs. E2AziRS expression is confirmed by anti-Ty1 staining. Without Azi in the growth medium, SAG1 is not detected due to the in-frame stop codon. Upon addition of Azi, robust expression of SAG1 is observed trafficking properly to the cell periphery. Red, rabbit anti-SAG1 antibody; green, mouse anti-Ty1 antibody. Scale bar represents 2 μm. (G) Western blot of whole cell lysates shows expression of the SAG1 nonsense mutant only when Azi is added to the medium. Wild-type RH parasites expressing endogenous SAG1 are used as a control. Azi, *p*-azidophenylalanine; E2AziRS, Azi-tRNA synthetase; HXGPRT, hypoxanthine-xanthine-guanine phosphoribosyl transferase; IFA, immunofluorescence assay; *SAG1*, surface antigen 1; UAA, unnatural amino acid; WT, wild-type.

In an amber suppression system, UAAs such as Azi are incorporated into an engineered amber stop codon (sequence TAG) within the protein sequence in lieu of premature termination (Fig 1A and 1B). To adapt this system to *T*. *gondii*, we generated a construct containing the Azi-specific enhanced *Escherichia coli* aminoacyl-tRNA synthetase (E2AziRS) [23] with a C-terminal Ty1 epitope tag driven by the *GRA1* promoter (Fig 1C). We also engineered an amber suppressor tRNA expression cassette driven by the *Toxoplasma* U6 promoter, which was recently characterized for use in the CRISPR/Cas9 system [24]. To maximize expression of the tRNA, we assembled three tandem copies of the cassette, a strategy that improves expression in mammalian systems [23]. Transfection of this construct into RHΔ*hxgprt* strain parasites showed that E2AziRS-Ty1 localizes to the cytoplasm, as expected (Fig 1D). We were able to generate stable lines expressing E2AziRS-Ty1, demonstrating that *T*. *gondii* tolerates constitutive expression of the aminoacyl-tRNA synthetase/tRNA cassettes.

To demonstrate that this system can be used to incorporate Azi into an endogenously translated protein, we explored the use of surface antigen 1 (SAG1), a highly abundant yet dispensable glycosylphosphatidylinositol (GPI)-anchored cell surface protein [25,26]. We built a SAG1 expression construct in which the second codon is mutated to the amber stop codon (SAG1 F2_TAG_, Fig 1E). We disrupted endogenous SAG1 using CRISPR/Cas9 and used this strain for expression of the aminoacyl-tRNA synthetase/tRNA cassettes and the SAG1 nonsense mutant [24]. In the absence of Azi, these parasites express E2AziRS-Ty1 but not SAG1 as observed by immunofluorescence assay (IFA, Fig 1F). However, upon overnight incubation in growth medium supplemented with Azi, robust expression of SAG1 is observed at the cell periphery. Quantification by western blot intensity shows approximately 35% SAG1 expression compared with wild-type parasites (Fig 1G). While the efficiency of incorporation is difficult to compare because the SAG1 amber mutant was driven from the *GRA1* promoter, these data demonstrate that Azi can be substantially incorporated into a control protein in the context of amber suppression similar to what is seen in other systems [23,27].

### Successful photoactivatable crosslinking using the UPRT homodimer

**Fig 2:**
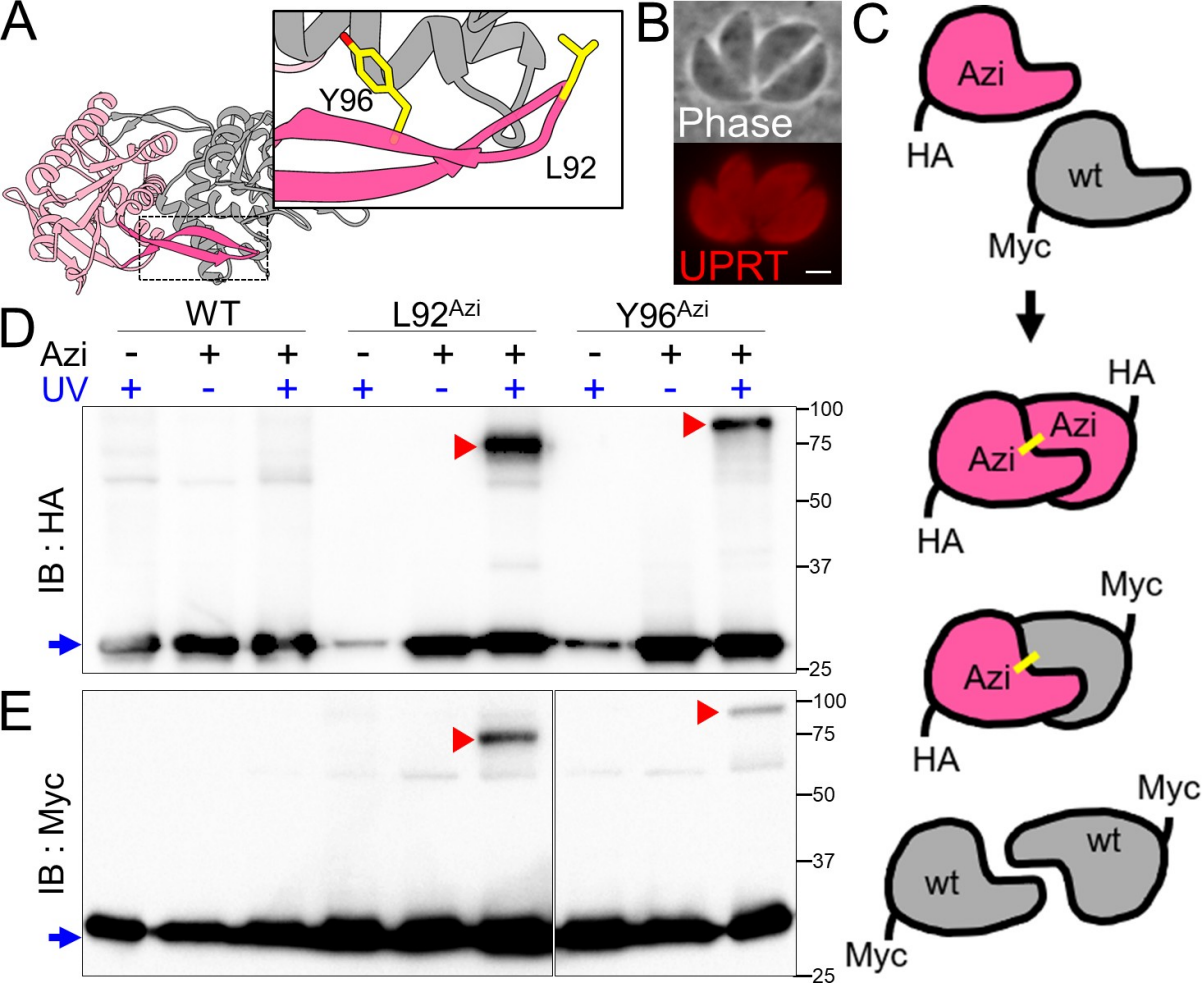
Site-specific crosslinking of UPRT in *T*. *gondii*. (A) The UPRT homodimer is stabilized by a β-arm (darker pink, structure adapted from PDB entry 1BD4). L92 and Y96 were chosen for Azi substitution based on orientation towards the other subunit in the crystal structure. (B) IFA showing cytoplasmic localization for parasites expressing the Y96 mutant UPRT-HA upon addition of Azi. Red, mouse anti-HA. Scale bar represents 2 μm. (C) Schematic for UPRT dimer formation using a second copy of UPRT with a Myc tag. UPRT proteins will assemble as either HA/HA or Myc/Myc homodimers, or a HA/Myc heterodimer. As the Myc-tagged monomers lack Azi, they should be crosslinked only when bound to an Azi-containing UPRT-HA partner. (D) Anti-HA western blot of UPRT crosslinking using strains expressing the synthetase/tRNA and either WT, L92, or Y96 UPRT-HA. Uncrosslinked UPRT migrates at 27 kDa (blue arrow). A small amount of UPRT is observed without Azi, indicating that nonspecific incorporation of other amino acids can occur, but this material is low abundance and lacks crosslinking ability. In the +Azi/+UV conditions, shifted products can be observed for both L92 and Y96 lines (red arrowheads), indicating successful crosslinking of a UPRT dimer. (E) Immunoblot of the UPRT crosslink samples with anti-Myc antibody verifies that the shifted products are covalently crosslinked UPRT homodimers. The Myc blot shows a lower relative efficiency of crosslinked to uncrosslinked material, presumably because the Myc-tagged monomers can only crosslink as the heterodimer, while the HA-tagged UPRT can crosslink as both the heterodimer and an HA/HA homodimer. Azi, *p*-azidophenylalanine; HA, hemagglutinin; IB, immunoblot; IFA, immunofluorescence assay; PDB, Protein Data Bank; UPRT, uracil phosphoribosyltransferase; WT, wild-type.

To determine if we could obtain UV-induced crosslinking of Azi in *Toxoplasma*, we employed the protein uracil phosphoribosyltransferase (UPRT), which forms a homodimer as revealed by the crystal structure (Protein Data Bank [PDB]: 1BD4, 1JLR) [28,29]. UPRT was also selected because it is a small cytoplasmic protein that is produced in abundance yet is not essential for parasite survival. The major region for dimer stabilization was reported to be a hydrophobic β-arm between residues 82 and 103. Within this region, we chose leucine 92 and tyrosine 96 for amber substitution (L92 and Y96) based on their polarity and orientation towards the partner subunit (Fig 2A). The L92 and Y96 mutants were engineered in a hemagglutinin (HA)-tagged UPRT construct, which localized to the cytoplasm upon addition of Azi to the media, as expected (Fig 2B). In addition, to demonstrate that any potential crosslinked products observed by western blot are indeed due to a covalently bound homodimer, we also expressed a Myc-tagged wild-type copy of UPRT, which should not be crosslinked unless bound to an activated Azi-containing UPRT-HA monomer (Fig 2C). To assess crosslinking, extracellular parasites were irradiated with 365-nm UV light, lysed in sample buffer for SDS-PAGE, and probed with anti-HA and anti-Myc antibodies. We observed upshifted signals corresponding to crosslinked species in both UPRT-HA Azi mutants, whereas the wild-type UPRT-HA control does not form appreciable signal other than nonspecific background (Fig 2D). The same upshifts are observed in the anti-Myc blot, indicating the products are bona fide crosslinked homodimers (Fig 2E). The discrepancy in migration between L92 and Y96 is likely to be a consequence of crosslinking to distinct regions of the partner subunit. The upshifts also do not correspond to a direct addition of constituent monomer masses but instead tend to reflect a larger mass, again likely due to the aberrant SDS-PAGE migration of the nonlinearly crosslinked peptides.

### Characterization of potential posttranslational modifications of ILP1

Prior to applying this crosslinking technology to ILP1, we first aligned the protein sequence from model apicomplexans and identified regions of interest that may be responsible for its trafficking and function (S1 Fig). The *Plasmodium* ortholog of ILP1 is named *Pf*G2 due to a conserved glycine at position 2 that is likely myristoylated and is essential for proper trafficking of the protein [18]. To assess whether this residue is similarly important in *T*. *gondii*, we mutated the second position glycine to alanine (G2A) and expressed it as a second copy in the parasite. ILP1 also contains two cysteine–cysteine motifs that are weakly predicted to be palmitoylated by CSS-PALM [30], and thus a quadruple C95S, C96S, C273S, C274S mutant (4Cys) was also generated. Surprisingly, both the G2 and 4Cys mutants appeared to correctly traffic to the IMC (S2B Fig). To determine if these sites were important for function, the endogenous copy of ILP1 was disrupted by CRISPR/Cas9 and the knockout was verified by IFA and PCR (S2A Fig). Quantification of plaque assays showed the 4Cys mutation did not have any significant effect on plaque formation, but the G2A mutant resulted in significantly smaller plaques, suggesting that myristoylation does play at least some role in ILP1 function (S2C Fig). *Pf*G2 expressed in *Toxoplasma* unexpectedly localized to the cytoplasm and attempts to knock out the endogenous ILP1 in this background were not successful, indicating that the *Plasmodium* ortholog cannot complement *Toxoplasma* ILP1 (S2B Fig).

### Application of the UAA system to ILP1 and preliminary identification of its partners

**Fig 3:**
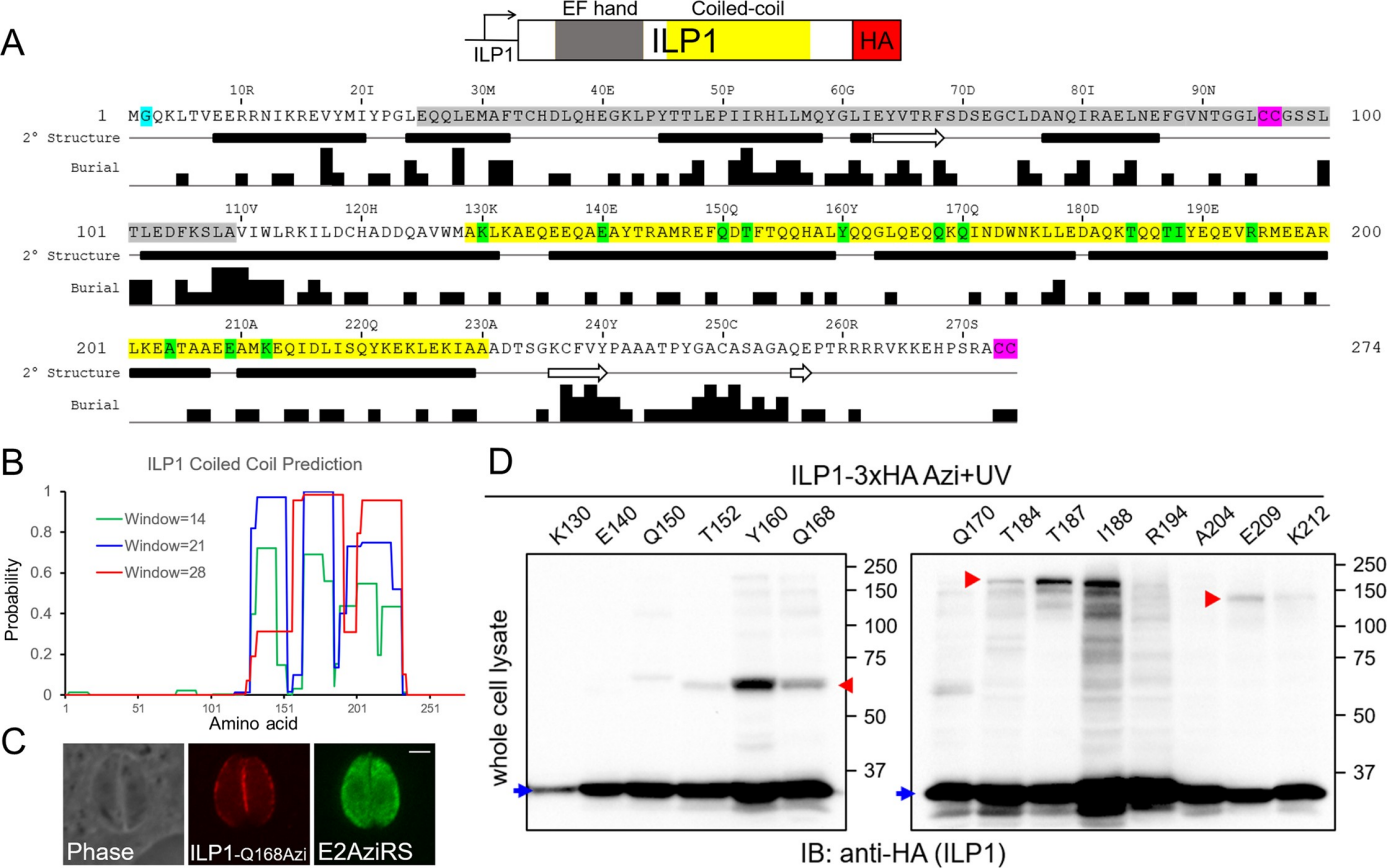
Site-specific crosslinking of ILP1 reveals multiple potential binding partners. (A) Diagram of ILP1 showing an N-terminal putative EF-hand domain (gray) followed by a coiled-coil domain (yellow). Also shown is the JPred secondary structure prediction of ILP1 revealing alpha-helices (black bars) and beta-strands (white arrows), as well as buried residue prediction used for choosing likely exposed residues for amber substitution [34]. Also noted are the potentially myristoylated glycine at position two (teal) and tandem cysteine prenylation/palmitoylation motifs internally and at the extreme C terminus (purple). Fourteen residues in ILP1 were chosen for amber mutagenesis to test for Azi-mediated crosslinking (green). (B) COILS prediction of ILP1; the window size refers to the number of residues used in the analysis. (C) Representative IFA of the Q168 ILP1 mutant containing Azi, which localizes properly to the parasite periphery. Red, rabbit anti-HA antibody; green, mouse anti-Ty1 antibody. Scale bar represents 2 μm. (D) Western blot of the ILP1 Azi mutants after UV irradiation reveals three major crosslinked species (red arrowheads). A smaller upshift (approximately 65 kDa) is observed for residues Y160 and Q168, with weak similar products for T152 and Q170. Residues T184, T187, and I188 exhibit a major band at approximately 200 kDa. E209 and K212 form a third upshift at approximately 140 kDa. Uncrosslinked ILP1 is denoted by the blue arrows (approximately 35 kDa). Azi, *p*-azidophenyalanine; E2AziRS, Azi-tRNA synthetase; HA, hemagglutinin; IB, immunoblot; IFA, immunofluorescence assay; ILP1, IMC localizing protein 1.

As the posttranslational modification sites were not critical for ILP1, we suspected that interaction with other components of the IMC cytoskeleton played an important role in function. Phyre2 analysis reveals a potential N-terminal EF hand-like domain from residues 25–109 (Fig 3A) [31]. However, this domain appears to be a degenerate EF-hand domain that is unlikely to bind calcium. Intriguingly, COILS analysis indicates a potential coiled-coil domain in the C-terminal region of the protein (residues 129–230) [32] (Fig 3B). Coiled-coil domains are alpha-helical assemblies that are involved in many protein binding–dependent functions such as vesicle transport and structural scaffolding, suggesting that this region may be involved in ILP1 function [33]. We first attempted co-immunoprecipitation (co-IP) experiments to determine putative targets of ILP1. Due to the detergent-insoluble nature of ILP1, we employed extensive sonication to disrupt the cytoskeleton and release ILP1 for co-IP [17]. Mass spectrometric analysis of the co-precipitated proteins revealed the IMC network-forming alveolins IMC1/3/4/6/10 as well as the glideosome-associated proteins MLC1 and GAP45 (S3 Fig). However, this approach was insufficient for determining direct partners, suggesting that our UAA approach might better reveal direct interactions of ILP1.

To apply the UAA system to ILP1, we focused on the coiled-coil domain of the protein and used secondary structure and residue burial predictions to guide the construction of 14 amber mutants (Fig 3C: K130, E140, Q150, T152, Y160, Q168, Q170, T184, T187, I188, R194, A204, E209, K212). Each of the mutants were stably expressed in parasites containing the aminoacyl-tRNA synthetase/tRNA cassettes, and the strains were subjected to Azi incorporation and photocrosslinking. Western blot analysis of the irradiated parasites resulted in six appreciable crosslinked upshifts (strains Y160, Q168, T184, T187, I188, and E209) that appeared to represent three distinct migration patterns at approximately 65 kDa, approximately 200 kDa, and approximately 140 kDa (Fig 3D).

### Identification of IMC3 as a binding partner of ILP1

**Fig 4:**
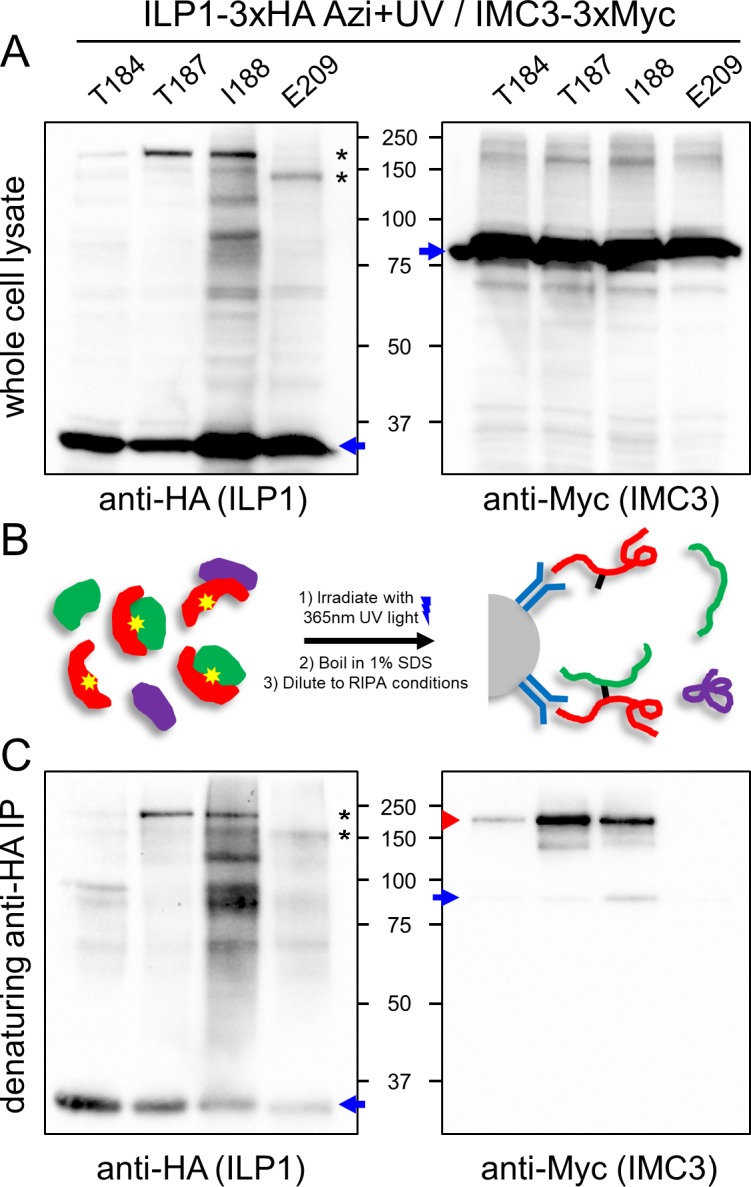
IMC3 is a direct binding partner of ILP1. (A) ILP1-3xHA amber mutants yielding the two large upshifted bands (T184, T187, I188, and E209) were expressed in an endogenously tagged IMC3-3xMyc background that also contains the synthetase/tRNA pair. Following Azi addition and UV irradiation, the uncrosslinked (blue arrow) and crosslinked species (black asterisks) can be reproduced in this strain. When probing for IMC3-3xMyc, uncrosslinked protein is observed (blue arrow), but a persistent high molecular weight background prevents confirmation of any potential upshift of IMC3. (B) Strategy for denaturing IP. Boiling in SDS disrupts the parasite’s cytoskeleton and protein–protein interactions of its components. The lysate is diluted to RIPA conditions for IP, and only the target protein (red) and covalently attached partners (green) are purified. (C) Western blot analysis of ILP1 denaturing IP recapitulates uncrosslinked (blue arrow) and crosslinked (black asterisks) ILP1 Azi mutants. As seen in the anti-Myc blot, the IP procedure eliminates the majority of the higher molecular weight background and reveals Myc-reactive crosslinked bands for residues T184, T187, and I188 (red arrowhead), demonstrating that IMC3 is indeed the partner at these residues. A small amount of uncrosslinked IMC3 that is likely to be reassociating with ILP1 is seen following dilution of the denatured lysate for IP (blue arrow). In contrast, no anti-Myc signal is seen for E209, indicating that this residue does not bind to IMC3. Azi, *p*-azidophenylalanine; HA, hemagglutinin; ILP1, IMC localizing protein 1; IP, immunoprecipitation.

To identify the shifted partners of ILP1, we investigated the possibility of a direct interaction between ILP1 and one of the alveolins, as several of these components of the IMC cytoskeleton were present in our immunoprecipitation (IP) data. IMC3, -6, and -10 are enriched in nascent daughter buds during endodyogeny similarly to ILP1 [35], and their sizes are consistent with the shifts observed for the two larger crosslinked products. However, IMC1 and IMC4 also represent good candidates even though these proteins are more equally present in daughter buds and maternal parasites. To determine if IMC3 was one of the larger products, we endogenously Myc-tagged this protein in the T184, T187, I188, and E209 UAA strains (Fig 4A). Unfortunately, potential crosslinked products in the western blot of whole cell lysates were not visible above the background, preventing us from determining if IMC3 was the shifted partner. We thus used a denaturing IP approach, in which the IMC cytoskeleton was first disrupted by boiling in 1% sodium dodecyl sulfate (SDS) and then diluted to standard radioimmunoprecipitation assay (RIPA) buffer conditions for IP (Fig 4B). We were able to purify both uncrosslinked ILP1 and the shifted products as assessed by anti-HA staining (Fig 4C). Probing the eluates with anti-Myc antibodies showed that IMC3 was indeed crosslinked to ILP1 in the T184, T187, and I188 strains but not the E209 strain. This result demonstrates that ILP1 binds to IMC3 in the IMC cytoskeleton and maps the IMC3 binding interface on ILP1.

### The C-terminal region of IMC3 is necessary for ILP1 crosslinking

**Fig 5:**
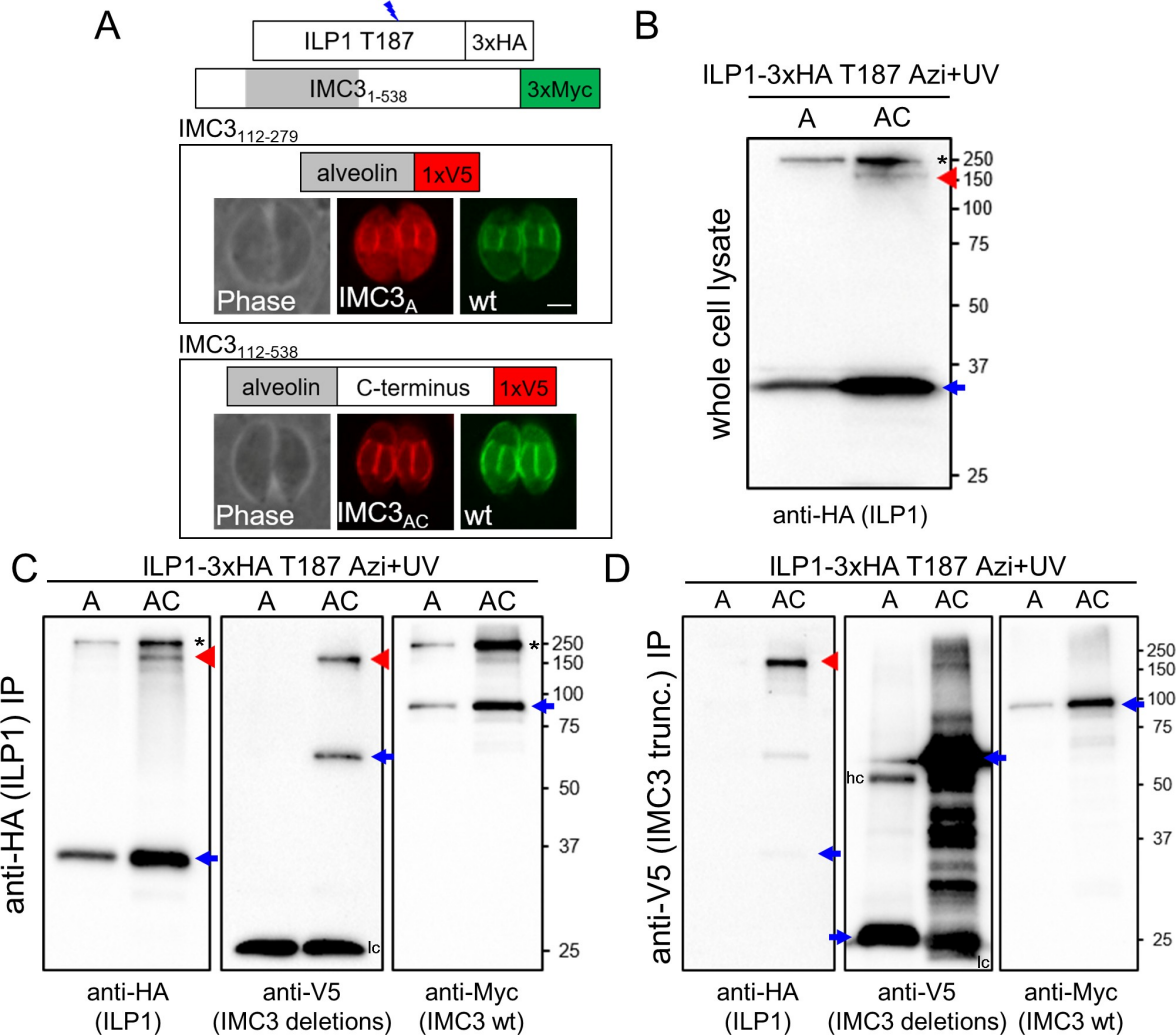
The C-terminal region of IMC3 is required for binding to ILP1 at T187. (A) Diagram and IFA of IMC3 truncations expressed in *Toxoplasma* to determine which region of IMC3 binds to ILP1 T187. In endogenously tagged IMC3-3xMyc parasites, regions corresponding to alveolin only (112–279, IMC3_A_) and alveolin plus C terminus (112–538, IMC3_AC_) were tagged with V5 and localized. IMC3_A_ partially mislocalizes to the maternal cytoplasm, suggesting that although the alveolin domain plays a role in IMC targeting, the inclusion of the C-terminal region of the protein improves IMC targeting similarly to wild-type IMC3. Red, mouse anti-V5 antibody; green, rabbit anti-Myc antibody. Scale bar represents 2 μm. (B) Western blot showing the high molecular weight product corresponding to a crosslinked full-length IMC3 in parasites expressing either IMC3_A_ or IMC3_AC_ (black asterisk). However, another smaller band (red arrowhead) is seen in the IMC3_AC_ lysate, likely representing an ILP1/IMC3_AC_ crosslinked product. The uncrosslinked ILP1 Azi mutant is denoted with a blue arrow. (C) ILP1-3xHA T187 denaturing IP shows the same ILP1 shifted products seen in whole cell lysates (first panel, black asterisk and red arrowhead), but an anti-V5 blot now clearly labels a band migrating at the same position as the new smaller anti-HA upshifted product (second panel, red arrowhead), demonstrating that this species corresponds to ILP1 T187 crosslinked to the IMC3_AC_. The light chain (lc) signal seen at 25 kDa obscures detection of residual uncrosslinked IMC3_A_. However, detecting the original ILP1 T187/IMC3 full length species in the anti-Myc blot (third panel, black asterisk) shows that IP in both IMC3_A_ and IMC3_AC_ conditions was successful. Uncrosslinked proteins are denoted with blue arrows. (D) Western blot analysis of anti-V5 denaturing IP performed with the same strains. Both IMC3_A_ and IMC3_AC_ are robustly enriched (second panel, blue arrows), but crosslinked ILP1 is only obtained in the IMC3_AC_ condition (first panel, red arrowhead). Some uncrosslinked IMC3-3xMyc is seen in the IP, likely reflecting interactions of this abundant alveolin in the lysate (third panel). Uncrosslinked proteins are denoted with blue arrows, and both heavy chains (hc) and lc are seen as extra signal in the anti-V5 blot. A, alveolin domain only; AC, alveolin domain and C-terminus; Azi, *p*-azidophenylalanine; HA, hemagglutinin; hc, heavy chain; IFA, immunofluorescence assay; ILP1, IMC localizing protein 1; IP, immunoprecipitation; lc, light chain; trunc., truncation.

We were interested in determining which region of IMC3 binds to ILP1 via residues T184, T187, and I188. The alveolin domain of IMC3 has been shown to be sufficient for targeting to the IMC, enabling this region to be tested as a binding partner using the UAA system [15]. We thus generated V5 epitope–tagged expression constructs of the alveolin domain alone (IMC3_A_) and the alveolin domain plus the C-terminal region of the protein (IMC3_AC_) to determine if these would crosslink to ILP1 (Fig 5A). While the IMC3 alveolin domain alone can direct the protein to the IMC as described, we also noticed some diffuse cytoplasmic staining. Complete trafficking to the IMC appears to be restored when the C-terminal region is included, demonstrating that this portion of the protein also plays a role in proper localization to the IMC. Upon Azi crosslinking in the T187 strain, IMC3_A_ did not form an additional shifted ILP1 product, indicating that this region is not sufficient for binding to T187 (Fig 5B). However, the IMC3_AC_ construct did result in a new smaller crosslinked product at the expected size for this IMC3 truncation. IP of ILP1 again showed the smaller product only in the IMC3_AC_ strain (Fig 5C). Probing with V5 revealed that this new shifted product is indeed IMC3_AC_. We additionally pulled down IMC3_AC_ using anti-V5 IP and observed that this co-precipitated crosslinked ILP1 (Fig 5D). This result suggests that ILP1 binds to the C-terminal region of IMC3, downstream of the alveolin domain.

### ILP1 crosslinks to IMC6 at residue E209

**Fig 6:**
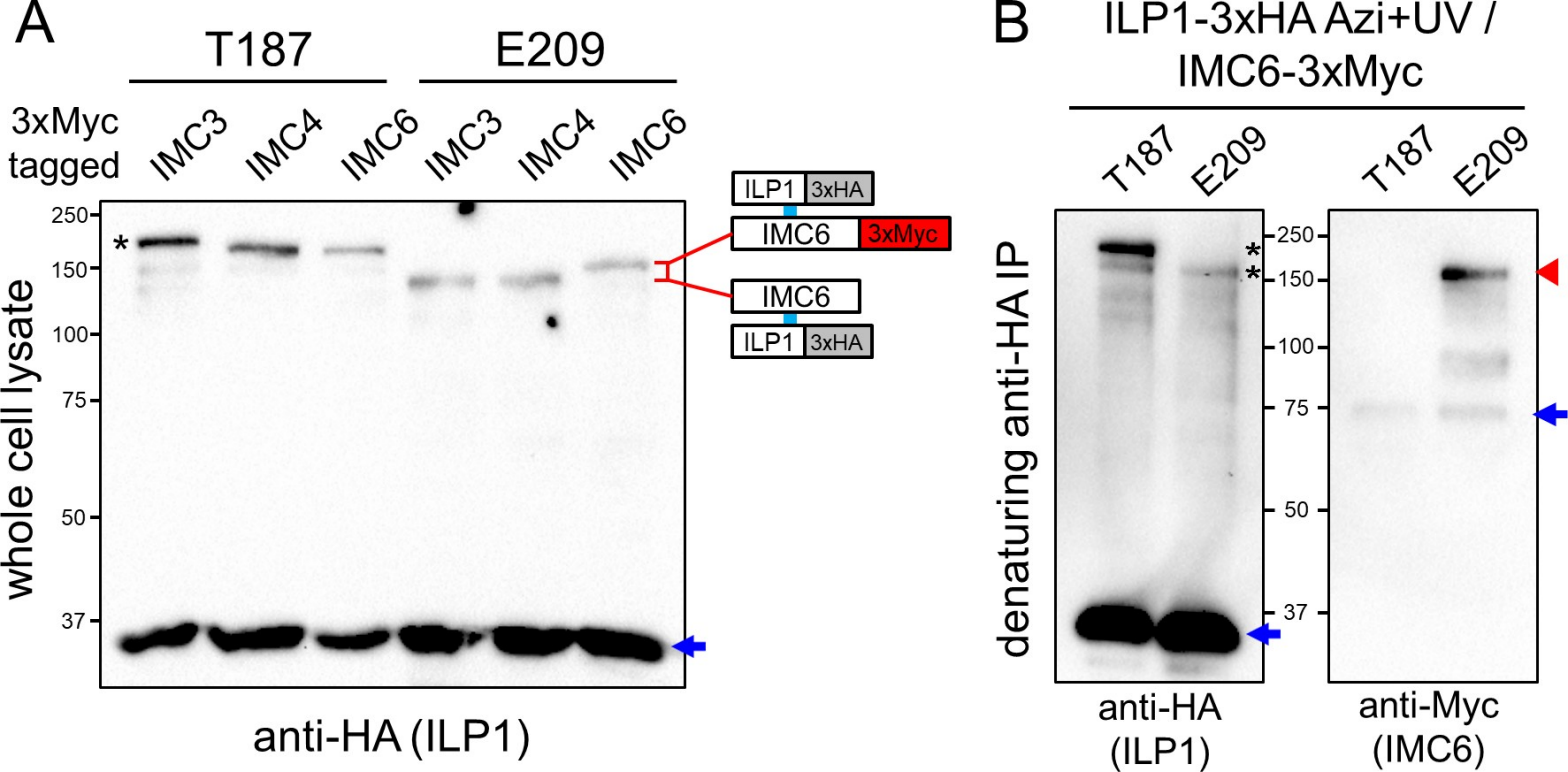
IMC6 is another direct binding partner of ILP1. (A) Crosslinking of T187 and E209 in strains endogenously 3xMyc-tagged for IMC3, IMC4 or IMC6. Tagging of IMC6 results in slower migration for the E209 Azi crosslinked product compared to the IMC3/4-3xMyc lines, which can be attributed to the addition of the epitope tag. A subtle shift can also be seen in the T187/ IMC3-3xMyc strain (black asterisk), as IMC3 is the partner at this residue. Uncrosslinked ILP1 Azi mutants are denoted with blue arrows. (B) Denaturing ILP1 co-IP to verify the E209/IMC6 interaction using IMC6 tagged parasites (T187, which binds IMC3, is used as a control). The anti-HA blot shows the expected uncrosslinked material (first panel, blue arrow) and crosslinked products (first panel, black asterisks) in the tagged lines, but only the E209 product is detected with anti-Myc, confirming the interaction with IMC6 (second panel, red arrowhead). Uncrosslinked IMC6, which was mostly removed by denaturation, is also present in both conditions (second panel, blue arrow). Azi, *p*-azidophenylalanine; co-IP, co-immunoprecipitation; HA, hemagglutinin; ILP1, IMC localizing protein 1; IMC, inner membrane complex.

To identify the protein crosslinked at residue E209, we examined the predicted sizes of the other alveolins for likely candidates. IMC4 and IMC6 have a smaller theoretical mass compared with IMC3, which may be reflected in the faster migrating upshift. We therefore tagged IMC4 and IMC6 with 3xMyc tags in the E209 strain and also in the T187 strain as a negative control (as this residue binds IMC3). Interestingly, the ILP1 E209 shifted product migrated slightly slower in the IMC6-3xMyc strain compared with those tagged for IMC3 and IMC4, indicating that IMC6 is likely the crosslinked partner (Fig 6A). In the T187 strains, a slight shift can also be detected when IMC3 was tagged, but the relative shift was less apparent due to the overall larger masses. Denaturing co-IPs were then performed to verify that IMC6 is the E209 crosslinked partner (Fig 6B). Anti-Myc staining demonstrated that E209 is indeed covalently crosslinked to IMC6, confirming this second interaction of ILP1 with an alveolin in the cytoskeleton.

### The N-terminal region of IMC6 is necessary for crosslinking to ILP1

**Fig 7:**
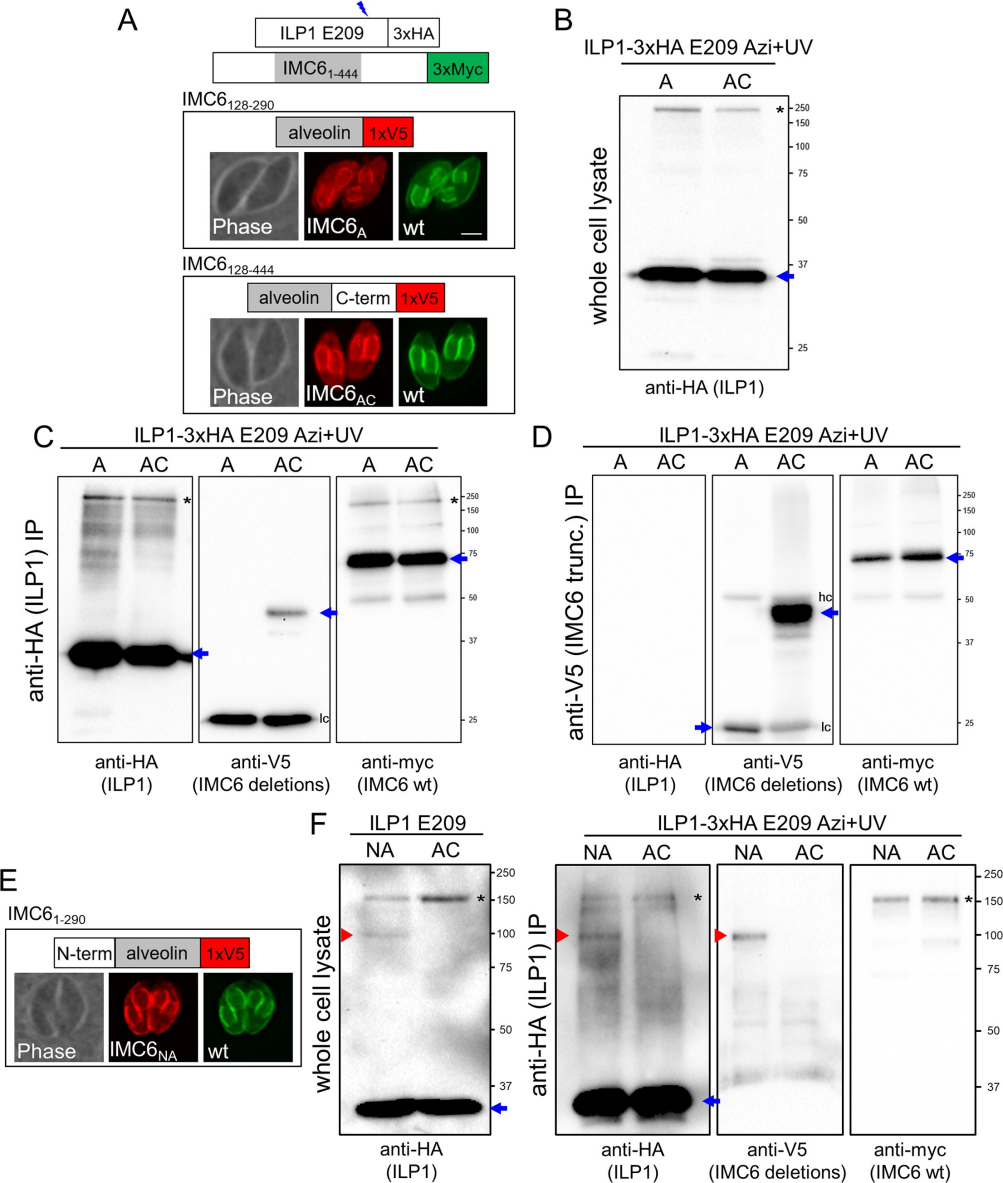
The N-terminal region of IMC6 is required for binding to ILP1 at E209. The experimental design mimics the one reported for IMC3 in Fig 6. (A) Alveolin only (128–290, IMC6_A_) and alveolin plus C terminus (128–444, IMC6_AC_) truncations tagged with a V5 epitope were visualized. Both truncations localize to the growing daughter IMC but also exhibit partial mislocalization in the maternal cytoplasm, which does not appear to be rescued with the addition of the C-terminal region. Red, mouse anti-V5 antibody; green, rabbit anti-Myc antibody. Scale bar represents 2 μm. (B) Western blot of whole cell lysate showing uncrosslinked ILP1 (blue arrow) and the original upshifted species (black asterisk) but no visible lower molecular weight band that would indicate crosslinking to the smaller size truncations. (C) ILP1-3xHA E209 denaturing IP enriches for ILP1 (first panel), but no upshift is observed in the anti-V5 blot (second panel), indicating that neither IMC6_A_ nor IMC6_AC_ is sufficient to crosslink to ILP1 at residue E209. The IMC6_A_ band is obscured by antibody light chain (lc) cross-reactivity. The anti-Myc blot (third panel) confirms successful IP, as seen by the original ILP1 E209/IMC6 full-length upshift (black asterisk). Uncrosslinked proteins are denoted with blue arrows. (D) Anti-V5 denaturing IP enriching for the IMC6 truncations correspondingly lacks any ILP1-3xHA signal (first panel). Uncrosslinked proteins are denoted with blue arrows, and both heavy chains (hc) and lc are seen as extra signal in the anti-V5 blot. (E) An N terminus plus alveolin domain truncation of IMC6 (1–290, IMC6_NA_) appears to rescue localization like wild-type IMC6. Red, mouse anti-V5 antibody; green, rabbit anti-Myc antibody. (F) Anti-HA immunoblot of both whole cell lysate (first panel, red arrowhead) and after anti-HA denaturing IP (second panel, red arrowhead) reveals a smaller upshifted species (approximately 90 kDa) for the IMC6_NA_ strain, suggesting that ILP1 E209 is crosslinking to this mutant. Anti-V5 blot confirms that IMC6_NA_ is detected at this molecular weight size (third panel, red arrowhead), demonstrating the identity of an ILP1 E209/IMC6_NA_ crosslink. Anti-Myc blot confirms successful co-IP of the original IMC6 upshift (fourth panel). Uncrosslinked ILP1 is denoted with blue arrows, and the original upshift corresponding to ILP1 crosslinked to full-length IMC6 is denoted with black asterisks. A, alveolin domain only; AC, alveolin domain and C-terminus; Azi, *p*-azidophenylalanine; co-IP, co-immunoprecipitation; HA, hemagglutinin; hc, heavy chain; ILP1, IMC localizing protein 1; IMC, inner membrane complex; IP, immunoprecipitation; lc, light chain; NA, N-terminus and alveolin domain; wt, wild-type.

Having shown that ILP1 binds to the C-terminal region of IMC3, we attempted similar domain mapping experiments with IMC6. We again generated V5-tagged constructs of the alveolin domain alone (IMC6_A_) or alveolin plus the C-terminal region (IMC6_AC_, Fig 7A). Curiously, the diffuse localization in the maternal cytoplasm occurred in both the IMC6_A_ and IMC6_AC_ strains. Neither of these truncations resulted in an additional crosslinked product, by western blot of either whole cell lysates or IPs (Fig 7B–7D). Instead, proper staining at the parasite periphery was observed when the N-terminal portion of IMC6 was added to the alveolin domain (IMC6_NA_, Fig 7E). ILP1 E209 Azi incorporation and photocrosslinking in this background resulted in a new upshifted product of the anticipated size for IMC6_NA_ (Fig 7F). This interaction was confirmed by denaturing IP as performed above. Together, these data indicate that the ILP1 binding site resides within the N-terminal region of IMC6.

### IMC27 is the third ILP1 binding partner

**Fig 8:**
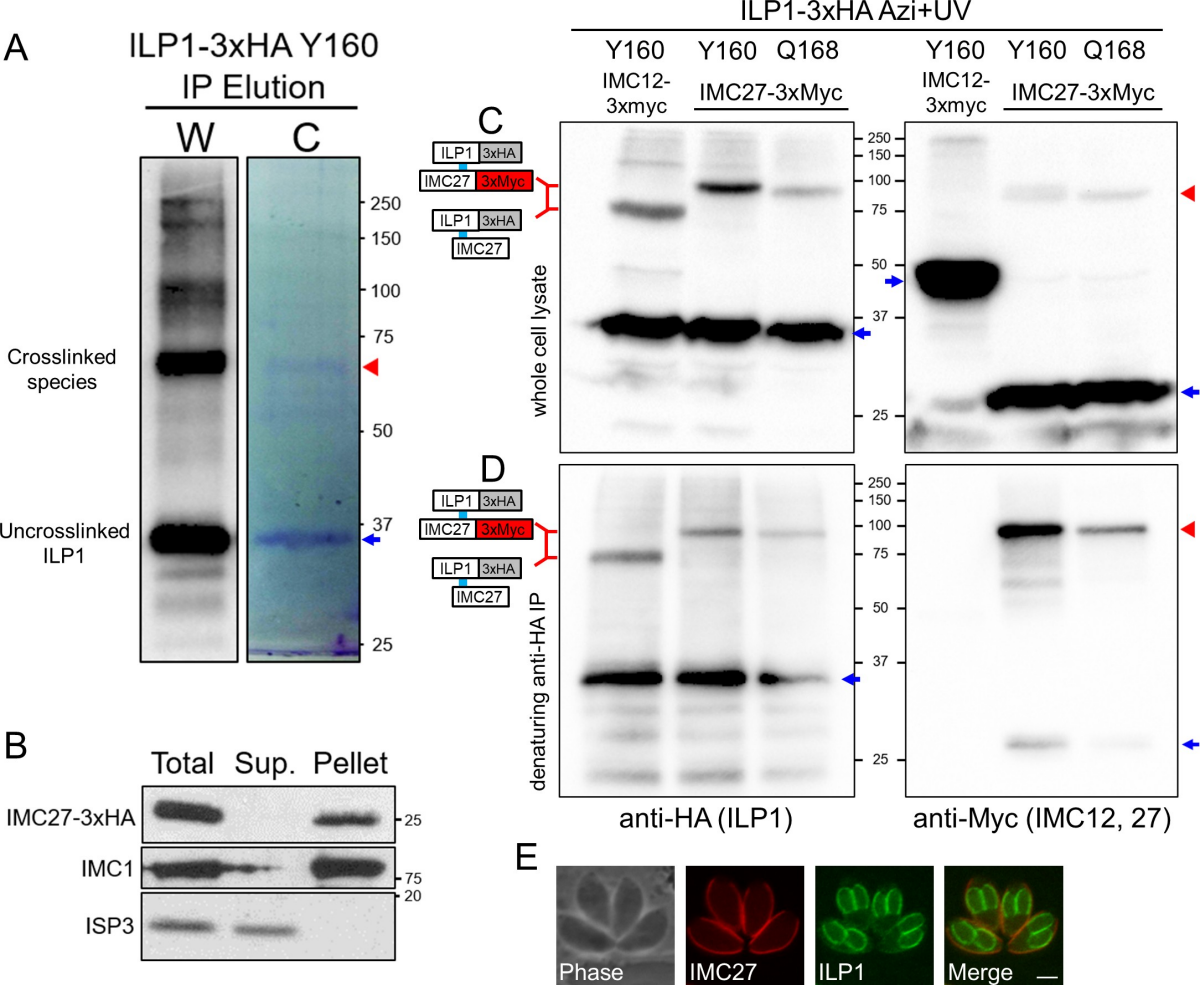
Mass spectrometric identification of crosslinked proteins reveals IMC27 as an ILP1 binding partner. (A) Western blot (W) and Coomassie gel (C) analyses of large-scale denaturing IP of Y160. The region of gel containing the crosslinked ILP1 species (red arrowhead) was excised and processed for LC-MS/MS peptide identification. Uncrosslinked ILP1 is denoted with a blue arrow. (B) Detergent fractionation showing that IMC27 is firmly associated with the IMC cytoskeleton, like ILP1 [17]. IMC1 is a control for the insoluble fraction whereas membrane-associated ISP3 is readily solubilizes upon detergent extraction. (C) Photocrosslinking of Y160 and Q168 in strains endogenously 3xMyc tagged for IMC27 (or IMC12 as a control). Tagging of IMC27 results in slower migration of the crosslinked product compared to that of the IMC12 tagged strain, indicating IMC27 is the partner. The shifted products are also detected in the anti-Myc blot (second panel, red arrowhead). Uncrosslinked proteins are denoted with blue arrows. (D) Denaturing IP shows the same pattern of shifted products for ILP1. Probing the samples with anti-Myc (for IMC29 or IMC12) confirms the higher migrating shifted product are IMC27 (red arrowhead), while uncrosslinked IMC27 is largely removed (blue arrow). IMC12-3xMyc is completely eliminated by denaturation. Uncrosslinked proteins are denoted with blue arrows. (E) IFA of IMC27-3xMyc parasites reveals that it localizes solely to the maternal IMC, indicating that interaction with ILP1 via Y160 and Q168 occurs within this subcompartment rather than the forming daughters. Red: mouse anti-Myc antibody, green: rat anti-ILP1 antibody. Scale bar represents 2 μm. Azi, *p*-azidophenylalanine; HA, hemagglutinin; IFA, immunofluorescence assay; ILP1, IMC localizing protein 1; IMC, inner membrane complex; IP, immunoprecipitation; ISP3, IMC subcompartment protein 3; LC-MS/MS, liquid chromatography tandem mass spectrometry.

None of the alveolins were candidates for the lower molecular weight product identified by Y160 and Q168, and thus we performed a large-scale crosslinking and IP experiment to identify the partner at these residues. As Y160 showed more robust crosslinking, this strain was expanded for a large-scale denaturing IP, and the eluted proteins were separated by SDS-PAGE and viewed by Coomassie staining (Fig 8A). Proteins within the gel slice corresponding to the upshifted species were identified by liquid chromatography tandem mass spectrometry (LC-MS/MS) (S1 Table). As expected, ILP1 was the top hit in the spectrum, and the second most abundant protein identified was IMC27, a protein we previously identified using in vivo biotinylation in the IMC [14]. Detergent fractionation revealed that IMC27 is a component of the IMC cytoskeleton similar to ILP1 (Fig 8B), and the protein migrates at approximately 26 kDa (including an approximate 5-kDa epitope tag), potentially agreeing with the observed crosslink size. To confirm this interaction, IMC27 was endogenously 3xMyc tagged, and this strain was transfected with the synthetase/tRNA and either ILP1 Y160 or Q168 constructs. IMC12 was also 3xMyc tagged in the Y160 strain as a negative control. Western blot analysis showed a distinct difference in migration between IMC27 tagged and untagged lines, indicating that IMC27 is the partner at these residues (Fig 8C). Denaturing IPs demonstrated that the shifted product stains with both HA (ILP1) and Myc (IMC27) antibodies, confirming that IMC27 is the crosslinked partner (Fig 8D). Intriguingly, while ILP1 and its partners IMC3 and IMC6 are all enriched in daughter buds, IMC27 is completely restricted to the maternal IMC (Fig 8E). Thus, the interaction between ILP1 and IMC27 is likely to only occur in maternal parasites following the completion of replication.

## Discussion

The implementation of a photoactivatable UAA crosslinking system in *T*. *gondii* enables a new tool for studying protein–protein interactions that govern parasite-specific biology. Mutant proteins containing the photoreactive UAA Azi are produced in vivo, ensuring proper folding and localization of target complexes in their native cellular environment. This avoids the disadvantages encountered in exogenous protein interaction techniques such as recombinant expression and yeast two-hybrid screens, in which artificial experimental conditions may disrupt legitimate associations or form false positives. Unlike more traditional chemical crosslinkers that employ a spacer arm, Azi is considered a zero-length crosslinker and therefore requires proper positioning of amber stop codon substitution near a binding interface to obtain crosslinking. While amber stop codon positioning can be challenging, successful crosslinking not only identifies the interacting partner but also maps the precise interaction domain on the bait protein, thereby providing more structural information of a complex compared to alternative protein–protein interaction approaches.

A common concern for expanding the genetic code for UAAs is the appropriation of a stop codon. When initially developed in *E*. *coli*, the system was designed to use the amber stop codon, as it is the least frequent (approximately 7%) of the three nonsense codons [36]. While *T*. *gondii* exhibits a more uniform stop codon distribution [37], we were able to obtain robust incorporation and reproducible crosslinking patterns with our proteins of interest, indicating that apprehensions regarding nonspecific incorporation or crosslinking are unwarranted. This is likely because Azi located at the C terminus of the majority of proteins would rarely reside in a binding interface required for the zero-length crosslinker. In addition, background derived from undesired Azi incorporation and photocrosslinking of endogenous proteins would not be observed by western blot unless these proteins were to somehow nonspecifically crosslink to our bait protein. A related concern is that Azi incorporation into endogenous proteins terminating with amber codons could result in the C-terminal extension of polypeptides during translation and disruption of proper termination. In our experience, prolonged exposure to Azi during growth does result in slower growth and ultimately cellular arrest, similar to prior observations using an alternative UAA [38], but this is not a significant issue, as the parasites are collected and processed within 24 hours of induction. Of the four proteins studied here (ILP1, IMC3, IMC6, and IMC27), the endogenous IMC3 gene terminates in an amber stop codon, but the effect of nonspecific Azi incorporation was not considered as this amber codon was changed to an ochre stop codon (TAA) in the process of C-terminal endogenous epitope tagging.

Our SAG1 and UPRT controls demonstrate efficient incorporation and crosslinking of Azi in target proteins of *T*. *gondii*. We initially chose an aromatic amino acid replacement in SAG1 (F2 to Azi) to limit any potential loss of function. However, our later experiments revealed that proteins generally appear to tolerate the single amino acid changes well, regardless of the properties of the residue being substituted. The risk of disrupting more critical residues is also mitigated by testing multiple mutants within a candidate interaction domain. To assess crosslinking, we utilized UPRT because of the available crystal structure and an understanding of its homodimer formation dynamics, guiding us toward the placement of amber codons at L92 and Y96. Both mutants generated SDS-PAGE upshifted products upon exposure to UV light, which were verified as homodimers using an alternatively tagged copy of UPRT. We observed that the migration of upshifted products is consistently slower than the combined mass of the partners and may vary depending on crosslinking site. Similar aberrations in migration in Azi crosslinked samples have been observed in other systems [22,23,39].

The challenges of studying protein–protein interactions in the IMC are reflected in our initial ILP1 co-IP, in which we identified several IMC proteins but were not able to determine meaningful interactions. The UAA system overcomes these challenges and enables determination of precise interactions within the rigid cytoskeletal meshwork of the IMC. The small size and distinct coiled-coil region of ILP1 provided a reasonable area to test for interactions via Azi crosslinking. None of the point mutations appeared to affect IMC trafficking, but differences in the efficiency of Azi incorporation among the different mutants were observed by western blot, suggesting that some substitutions are better tolerated than others (Fig 3). As the alveolins are believed to compose the cytoskeletal foundation through formation of intermediate filament-like polymers, those that shared similar localization patterns to ILP1 (IMC3/6/10) were top candidates for binding partners. We demonstrated direct crosslinking of ILP1 to both IMC3 and IMC6, providing the first insight into the organization of the alveolar network. When verifying these interactions, we found that the denaturing co-IP procedure was particularly useful for reducing nonspecific background and also dramatically reducing undesirable amounts of uncrosslinked prey that otherwise confounded our western blot results. Like UPRT, the ILP1 upshifts consistently migrated slower than anticipated, but unlike the control, the crosslink clusters seemed to migrate similarly, possibly due to the linear nature of the coiled-coil domain. The binding sites on ILP1 are relatively close to each other, and whether one ILP1 molecule can simultaneously bind to both partners remains unclear. We constructed a T187/E209 double Azi mutant in an attempt to determine if a trimeric complex was possible, but lower UAA incorporation in this mutant and the large expected mass made the results inconclusive.

Our IMC3 and IMC6 deletion analyses also demonstrated that the C-terminal region of IMC3 downstream of the alveolin domain and the N-terminal region of IMC6 upstream of the alveolin domain are necessary for binding to ILP1. The improved localization to the IMC upon inclusion of these regions suggests that interaction with ILP1 enhances its association within the network. While these results strongly suggest that ILP1 binds to these domains of IMC3/6, we cannot exclude the possibility that our deletions alter how the proteins interact. Additional truncations of IMC3/6 are likely to isolate a sufficiently small region that would enable precise determination of the corresponding binding regions on IMC3 and IMC6 using the UAA system.

IMC27 was identified as the binding partner at residues Y160/Q168 of ILP1 using a large-scale IP and LC-MS/MS peptide identification approach. Our success demonstrates that even though the abundance of crosslinked material is a fraction of the total amount of the purified bait protein, we can obtain yields that are adequate for mass spectrometric analysis. We explored the use of software such as Crossfinder, a tool for finding crosslinked peptides in an LC-MS/MS spectrum, to attempt to map the binding location of the prey, but this was unsuccessful [40]. This may be due to a particularly large region of IMC27 (residues 96–141, 28% of the total length) that lacks tryptic cleavage sites. Intriguingly, IMC27 localizes solely to the maternal IMC, indicating that there is a transition where ILP1 forms new interactions following the maturation of the budding daughter IMC. Because our experiments were carried out using extracellular parasites, the binding of IMC3 and IMC6 are also likely occurring in the maternal IMC. However, the precise colocalization of ILP1, IMC3, and IMC6 throughout the cell cycle suggests that these associations are likely to occur in both the maternal and daughter IMC. Future experiments comparing intracellular and extracellular parasites will help distinguish events that occur in the daughter and maternal IMC.

The interactions of ILP1 and its binding partners also begin to unravel how each are differently utilized in the *Toxoplasma* and *Plasmodium* IMC. In *Toxoplasma*, ILP1 is essential, and its alveolin partners IMC3 and IMC6 are predicted by genome-wide CRISPR studies to be very important or essential as well [41]. In contrast, both *Pf*G2 and *Pf*IMC1h (*Toxoplasma* IMC3) can be disrupted, resulting in similar morphological changes in ookinetes and sporozoites that suggest these partners play important, but not essential, roles in organizing the IMC cytoskeleton [18,42]. Unlike IMC3, IMC6 (*Pf*IMC1k) and IMC10 (*Pf*IMC1j) appear to be essential or important for growth in both apicomplexans [43], indicating a more conserved role in maintaining IMC integrity. Interestingly, *Toxoplasma* IMC27 is predicted to be dispensable, while its Plasmodial ortholog PF3D7_0518900 is considered to be either essential or important for growth, suggesting that there is additional functional divergence in the IMC between these apicomplexans. Overall, these comparisons indicate that *Toxoplasma* relies on ILP1 and its binding partners for structural support of the IMC to a greater degree compared with *Plasmodium*.

To our knowledge, this is the first use of photoreactive crosslinkers by expansion of the genetic code in any protozoan. We have demonstrated that site-specific crosslinking using Azi can be used to decipher the interactions of ILP1 within the IMC, and we anticipate successful application towards other IMC proteins, such as the alveolins, to further determine the organization of this organelle. Intriguingly, a recent proteomic analysis of the microtubule-based conoid has revealed that many of its constituent proteins have coiled-coil domains, making this compartment another good candidate for our system [44]. Alternative regions of ILP1, such as the N-terminal EF-hand domain, are also open for investigation. Assuming the UAA system is applicable to other protozoans, photoreactive crosslinkers also would be excellent tools for probing other unique structures such as the flagellar pocket of trypanosomes, the axostyle of trichomonads, or the ventral disc of *Giardia* [45–49].

## Materials and methods

### *T*. *gondii* and host cell culture

Parental *T*. *gondii* RHΔ*hxgprt* and subsequent strains were grown on confluent monolayers of human foreskin fibroblasts (HFFs, ATCC, Manassas, VA) at 37°C and 5% CO_2_ in Dulbecco’s Modified Eagle Medium (DMEM) supplemented with 5% fetal bovine serum (Gibco), 5% Cosmic calf serum (Hyclone), and 1× penicillin-streptomycin-L-glutamine (Gibco). Constructs containing selectable markers were selected using 1 μM pyrimethamine (dihydrofolate reductase-thymidylate synthase [DHFR-TS]), 50 μg/mL mycophenolic acid-xanthine (HXGPRT), or 40 μM chloramphenicol (CAT) [50–52]. Homologous recombination to the UPRT locus was negatively selected using 5 μM 5-fluorodeoxyuridine (FUDR) [53].

### Plasmid construction and mutagenesis

Sequences for E2AziRS and cognate amber suppressor tRNA (*Bst*-Yam) were obtained from pIre-Azi3 [22,23]. Primers P1/P2 were used to amplify E2AziRS with NsiI/PacI overhangs and a C-terminal Ty1 epitope tag. This product was ligated into pGRA-HA-HPT [54] to drive constitutive expression from the *GRA1* promoter and enable stable integration with HXGPRT (pGra-E2AziTy.HPT). The tRNA cassette was generated by synthesizing a gBlock gene fragment (IDT, San Diego, CA) consisting of the 82-bp *Bst*-Yam tRNA sequence flanked by a portion of the *T*. *gondii* U6 promoter and poly-thymine RNA polymerase III terminator as described [24], with AvrII/XbaI restriction sites (S1 Text). This insert was ligated into AvrII/XbaI-digested pU6-Universal to restore the complete U6 cassette, replacing the CRISPR/Cas9 gRNA scaffold with *Bst*-Yam. The cassette (TgU6-tRNA_CUA_) was amplified with P3/P4 to incorporate 5′ Acc65I-XhoI and 3′ SalI-BglII-XbaI restriction sites and subcloned into the pJET1.2/blunt vector (ThermoFisher). Triple tandem cassettes were constructed as previously described [23]. Briefly, a XhoI/SalI-flanked TgU6-tRNA_CUA_ insert was ligated into a SalI-linearized TgU6-tRNA_CUA_ pJET vector using complementary ends. This was iterated to double and triple tandem copies of the tRNA cassette, confirming orientation of each cassette by diagnostic digests. The triple cassette was excised with Acc65I/BglII and ligated into pGra-E2AziTy.HPT to obtain the final construct pGra-E2AziTy.HPT.tRNAx3.

The following plasmids were all generated in a similar fashion by ligating a gene of interest using NsiI/PacI overhangs into a modified pGRA-HA-HPT backbone [54]. First, HXGPRT was replaced with a DHFR cassette amplified from p3xHA.LIC-DHFR [55,56] using primers P5/P6 and cloning via HindIII/NgoMIV. *SAG1* was amplified from RH genomic DNA using primers P7/P8, which flanked the gene with NsiI/PacI sites and mutated the second codon TTT to the amber stop codon TAG, which appears as the third codon due to the NsiI site (the correct start of the gene begins with the sequence MFPKAV …). The *UPRT* coding sequence was amplified from RH cDNA either with an N-terminal Myc tag (P9/P10) or a C-terminal HA tag (P11/P12) and ligated to obtain pGRA-Myc-UPRT_wt.DHFR and pGRA-UPRT-HA_wt.DHFR. Amber mutations L92* and Y96* were generated in pGRA-UPRT-HA_wt.DHFR using the Q5 Site Directed Mutagenesis kit (NEB, Ipswich, MA) using primers P13/P14 and P15/P16. The mutant UPRT-HA cassettes were amplified with NotI/PciI flanks (P17/P18) and ligated into pJET1.2/blunt. The cassette was excised with NotI and ligated into NotI-linearized pGRA-Myc-UPRT_wt.DHFR to obtain the final double Myc-UPRT/UPRT-HA expression vectors.

An ILP1 expression construct was assembled, with the endogenous promoter driving *ILP1* with a C-terminal 3xHA epitope tag and a DHFR marker (pILP1-3xHA_wt.DHFR). ILP1 amber mutants were generated using this parent vector, using the Q5 Mutagenesis kit and primers P19–46.

For IMC3 and IMC6 truncations, a UPRT locus knockout plasmid template with an ILP1 promoter and V5 C-terminal epitope was used for Gibson assembly. This vector was amplified with Q5 Hot Start polymerase (NEB, Ipswich, MA) using primers P47/P48. Coding sequences of the IMC3 and IMC6 truncations were amplified with Q5 polymerase using the online NEBuilder (https://nebuilder.neb.com) tool to append compatible Gibson overhangs. P49/P50 were used for amplifying IMC3 alveolin only, P49/P51 for IMC3 alveolin and C terminus, P52/P53 for IMC6 alveolin only, P52/P54 for IMC6 alveolin and C terminus, and P53/P55 for IMC6 N terminus and alveolin domains. Purified amplicons were used to generate the final constructs using the NEBuilder HiFi DNA Assembly kit (NEB, Ipswich, MA). The plasmids were linearized using DraIII or XmnI (NEB), transfected into endogenously tagged IMC3-3xMyc or IMC6-3xMyc RHΔ*hxgprt* parasites, and selected for recombination at the UPRT locus using FUDR.

The ToxoDB gene numbers used in this study are ILP1 (TgGT1_313380), SAG1 (TgGT1_233460), GRA1 (TgGT1_270250), UPRT (TgGT1_312480), IMC3 (TgGT1_216000), IMC4 (TgGT1_231630), IMC6 (TgGT1_220270), and IMC27 (TgGT1_259630). The PfG2 gene is PF3D7_0929600 on PlasmoDB.

### Antibodies

The HA epitope was detected with mouse monoclonal antibody (mAb) HA.11 (BioLegend, San Diego, CA) or rabbit polyclonal antibody (pAb) anti-HA (ThermoFisher). The Ty1 epitope was detected with mouse mAb BB2 [57]. The c-Myc epitope was detected with mouse mAb 9E10 [58] or rabbit pAb anti-Myc (ThermoFisher). The V5 epitope was detected with mouse mAb anti-V5 (ThermoFisher). *Toxoplasma*-specific antibodies include rabbit pAb anti-SAG1 [59], mouse mAb anti-IMC1 [60], mouse pAb anti-ISP3 [61], and mouse mAb anti-ROP7 [62]. Production of rat pAb anti-ILP1 and rabbit pAb anti-IMC6 is described below.

### Immunofluorescence assay and western blot

HFF were grown to confluence on glass coverslips and infected with *T*. *gondii*. After 18–36 hours, the coverslips were fixed with either 3.7% formaldehyde in PBS or 100% methanol and processed for immunofluorescence as described [63]. Primary antibodies were detected by species-specific secondary antibodies conjugated to Alexa Fluor 488/594 (ThermoFisher). Coverslips were mounted in Vectashield (Vector Labs, Burlingame, CA) and viewed with an Axio Imager.Z1 fluorescent microscope (Zeiss).

For western blot, parasites were lysed in 1× Laemmli sample buffer with 100 mM DTT and boiled at 100°C for 10 minutes. Lysates were resolved by SDS-PAGE and transferred to nitrocellulose membranes, and proteins were detected with the appropriate primary antibody and corresponding secondary antibody conjugated to horseradish peroxidase. Chemiluminescence was induced using the SuperSignal West Pico substrate (Pierce) and imaged on a ChemiDoc XRS+ (Bio-Rad, Hercules, CA). Quantification of western blot signal was performed with Image Lab software.

### Detergent extraction assay

Detergent solubility of IMC27 was assessed as previously described [13]. Briefly, IMC27-3xHA tagged parasites were collected and lysed in a 1% Triton X-100, 50 mM Tris-HCl, pH 7.4, 150 mM NaCl buffer supplemented with Complete Protease Inhibitor Cocktail (Roche) and incubated on ice for 30 minutes. Lysates were centrifuged, and equivalent loads of the total, supernatant, and pellet samples were run on SDS-PAGE and immunoblotted, using IMC1 as the insoluble control and ISP3 as the soluble control.

### Endogenous epitope tagging of genes of interest

For C-terminal endogenous tagging, a pU6-Universal plasmid containing a protospacer against the 3′ UTR of the gene of interest approximately 100 bp downstream of the stop codon was generated as previously described [24]. A homology directed repair (HDR) template was PCR-amplified using the *Δku80*-dependent LIC vectors (e.g., p3xHA.LIC-DHFR, p3xMyc.LIC-CAT) that includes the epitope tag, 3′ UTR, and selection cassette. The 60-bp primers include 40 bp of homology immediately upstream of the stop codon or 40 bp of homology within the 3′ UTR downstream of the CRISPR/Cas9 cut site. This template was amplified in a total of 400 μL, purified by phenol-chloroform extraction, precipitated in ethanol, and electroporated into RH*Δhxgprt* parasites, along with 100 μg of the sequence-verified pU6-Universal plasmid. Transfected cells were allowed to invade a confluent monolayer of HFF, and appropriate selection was applied the following day. Successful tagging was monitored by IFA, and clonal lines of properly tagged parasites were obtained through limiting dilution. IMC3 was C-terminally tagged with this process using gRNA and HDR primers P56–59, IMC4 using P60–63, IMC6 using P64–P67, IMC12 using P68–71, and IMC27 using P72–75.

### In vivo photocrosslinking of protein complexes

Parasites expressing the synthetase/tRNA cassette and mutant protein of interest constructs were allowed to infect HFFs overnight at a multiplicity of infection of approximately 3 and the growth medium was replaced with fresh medium supplemented with 1 mM Azi (Bachem, Torrance, CA). Following a 24-hour incubation period and lysis of the host cells, extracellular parasites were collected by centrifugation and resuspended in an adequate volume of PBS for UV irradiation (approximately 10^7^ parasites per mL of PBS, in tissue culture plates). The plates containing resuspended parasites were floated on an iced water bath and placed without lids in a Spectrolinker XL-1000 UV crosslinker (Spectroline, Westbury, NY) equipped with 365-nm (UV-A) bulbs. Parasites were irradiated for 20 minutes with periodic mixing using a micropipette. The cells were then collected by centrifugation and lysed for either co-IP or directly in sample buffer for SDS-PAGE.

### co-IP

Traditional co-IP was carried out as previously described [61]. For denaturing co-IP of crosslinked proteins, irradiated parasites were lysed in a 1% SDS/50 mM Tris, pH 8.0/150 mM NaCl buffer and boiled at 100°C for 10 minutes to completely denature protein complexes. The lysate was centrifuged, and the supernatant was diluted 10-fold to RIPA conditions prior to IP. Precipitated proteins are either eluted in sample buffer or by high pH with a 100 mM triethylamine solution and dried using a vacuum concentrator. Colloidal Coomassie staining was accomplished using GelCode Blue Stain (ThermoScientific). Gel slices were excised and processed for mass spectrometry. IPs were performed using rat anti-HA (Roche) or mouse anti-V5 (Sigma) agarose beads.

### Characterization of ILP1 acylation mutants and *Pf*G2 complementation

ILP1 acylation mutants were generated using pILP1-3xHA_wt.DHFR as a template. For the G2A mutant, the ILP1 promoter and N-terminal region were amplified using primers P76/P77, incorporating the desired mutation. This fragment was ligated using HpaI/HindIII to the vector digested with EcoRV/HindIII. For the 4Cys mutation, the C95S, C96S mutant was made using the Q5 Site-Directed Mutagenesis kit with primers P78/P79. The 4Cys mutant was then constructed by amplifying the C95S, C96S template with primers P80/P81, which incorporated the C-terminal C273S and C274 mutations, and cloned using HpaI/NotI. The codon-optimized *Pf*G2 coding sequence gBlock (S1 Text) was synthesized and incorporated into an ILP1 promoter expression construct.

The HA epitope tag in pUPRTKO-HA plasmid [64] was replaced with a V5 tag by digesting the vector with NotI/PacI and ligating NotI-V5-PacI annealed oligos P82/P83. This vector was then digested with NheI/NotI, and the ILP1 promoter along with the wild-type, G2A, and 4Cys ILP1 mutants or *Pf*G2 gene were ligated with the same sites. The final pUPRTKO-V5 constructs were linearized with DraIII and transfected into ILP1-3xHA DHFR RH*ΔhxgprtΔku80* parasites, and expression of the mutants was confirmed by IFA. Clonal lines were then transfected with an NcLiv *GRA7*–driven HXGPRT HDR knockout template with flanking homology to the endogenous ILP1 locus [14], and the pU6-Universal plasmid was transfected against a protospacer within the first intron of the genomic sequence (P84–P87). After selection, loss of endogenous ILP1-3xHA expression was confirmed by IFA. Genomic DNA was extracted using the PureLink Genomic DNA kit (Invitrogen), and successful knockouts were confirmed by PCR using P88/P89 spanning two introns and P90/P91 spanning the ILP1 promoter/NcGra7 promoter interface.

Plaque assay of the ILP1 complementation mutants was performed as previously described [65]. Six-well plates were seeded with HFF and allowed to reach confluency. A total of 100–600 parasites were added per well and allowed to grow for 7 days. The monolayers were fixed with 100% methanol for 3 minutes, washed with PBS, and stained for visualization. The areas of 50 plaques per condition were quantified with the ZEN 2 software (Zeiss). Significance levels were calculated by unpaired *t* test.

### Antibody production

The complete coding sequences for ILP1 and IMC6 were cloned into the pET His6 TEV LIC bacterial expression vector (a gift from Scott Gradia, Addgene plasmid #29653) using primers P92–95. The constructs were transformed into BL21(DE3) *E*. *coli*, and proteins were induced with 1 mM IPTG and purified using Ni-NTA agarose under denaturing conditions as described [63]. The samples were then dialyzed into PBS to remove the urea, and rat or rabbit antisera were produced by Cocalico Biologicals (Stevens, PA).

### Tandem mass spectrometry

The protein mixtures were reduced, alkylated, and digested by the sequential addition of trypsin and Lys-C proteases. Samples were then desalted using Pierce C18 tips, eluted in 40% acetonitrile, and dried and resuspended in 5% formic acid. Desalted samples were separated on C18 reversed phase (1.9 μM, 100-Å pores, Dr. Maisch GmbH, Germany) columns, packed with 25 cm of resin in a 75 μM inner diameter fused silica capillary. Digested peptides were fractionated online using a 140-minute water-acetonitrile gradient with 3% DMSO ionized using electrospray ionization by application of a distal 2.2 kV.

Upon electrospray ionization at 2.2 kV, ionized peptides were interrogated via tandem mass spectrometry (MS/MS) in a Thermo Orbitrap Fusion Lumos. For discovery acquisitions, data-dependent acquisition (DDA) was utilized with an MS1 scan resolution of 120,000 and MS2 resolution of 15,000 and a cycle time of 3 seconds. Data analysis was performed using the Integrated Proteomics Pipeline 2 (Integrated Proteomics Applications, San Diego, CA). MS/MS spectra were searched using the ProLuCID algorithm, and peptide-to-spectrum matches (PSMs) were organized and filtered based on a decoy database-estimated false discovery rate of <1% using the DTASelect algorithm. Database searching was performed using a FASTA protein database containing *T*. *gondii* GT1 translated ORFs downloaded from ToxoDB on February 23, 2016. Label-free intensity-based quantitation (LFQ) of the LC-MS/MS data was carried out by MS1 feature detection using chromatographic peak areas for peptide abundance through MaxQuant software package *v*.1.6.3.3 [66].

## Supporting information

S1 Fig Alignment of ILP1 in representative apicomplexans. ILP1 is well conserved in coccidians, while the *Plasmodium falciparum* ortholog is more divergent. The likely myristoylated glycine at position 2 is conserved. Two putatively palmitoylated cysteine pairs are present in the *Toxoplasma* sequence at residues C95, C96 and C273, C274. The internal pair is present in *Plasmodium* but missing in *Eimeria*, while the C-terminal pair is conserved in coccidians but only present as a single cysteine in *Plasmodium*. Alignment was generated using Clustal Omega [67] and shaded using BoxShade (https://embnet.vital-it.ch/software/BOX_form.html). Black highlights represent identity; gray highlights represent similarity. ILP1, IMC localizing protein 1
(DOCX)

S2 Fig Mutation of ILP1 putative posttranslational modifications and *Plasmodium* G2 localization. Loss-of-function mutations of the putative myristoylation (G2A) and palmitoylation (4Cys) sites were assessed by knocking in mutant copies to the UPRT locus and disrupting the endogenous ILP1 locus using CRISPR/Cas9. (A) Strategy and PCR analysis of endogenous ILP1 disruption. Knockouts were assessed by absence of amplification using intronic primers and positive amplification of the sequence between the ILP1 promoter and the NcGra7 promoter following HDR. (B) IFA showing that exogenous ILP1 WT, G2A, and 4Cys copies all localize normally to the parasite periphery. The *Plasmodium* G2 ortholog fails to localize properly and could not compensate for the endogenous ILP1 knockout. Red, mouse anti-V5 antibody; green, rabbit anti-IMC6 antibody. Scale bars represent 2 μm. (C) Plaque assays of the ILP1 mutants following knockout of endogenous ILP1. The ILP1 G2A mutant has a slight but significant growth defect when compared with the ILP1 WT strain (an approximate 50% reduction). The 4Cys mutant does not have any growth disadvantage compared with control. HDR, homology directed repair; IFA, immunofluorescence assay; ILP1, IMC localizing protein 1; IMC, inner membrane complex; NcGra7, *Neospora caninum Gra7* promoter; UPRT, uracil phosphoribosyltransferase; WT, wild-type; 4Cys, quadruple C95S, C96S, C273S, C274S mutant
(TIF)

S3 Fig Co-IP of ILP1 yields several known IMC proteins. (A) Representative silver stain of an anti-HA IP of ILP1-3xHA parasites performed after fractionation in 1% Triton-X 100 and extensive sonication of the pellet to solubilize the IMC cytoskeleton. RH parasites were used as a control. A gel slice containing a unique band (blue arrow) was excised and proteins were identified by mass spectrometry. Identified proteins included the alveolins and components of the glideosome. co-IP, co-immunoprecipitation; HA, hemagglutinin; ILP1, IMC localizing protein 1; IMC, inner membrane complex
(TIF)

S1 Table MaxQuant intensities of upshifted ILP1-Y160 band. Top 30 protein intensities calculated by MaxQuant of the excised band following ILP1-Y160 crosslinking and large-scale anti-HA IP. ILP1 and IMC27 are the top two proteins that are identified. HA, hemagglutinin; ILP1, IMC localizing protein 1; IMC, inner membrane complex; IP, immunoprecipitation
(XLSX)

S2 Table List of primers used in this study. (XLSX)

S1 Text List of synthesized gene fragments used in this study. (DOCX)

S1 Raw images Raw western blot and gel images. (PDF)

## Abbreviations

Azi: *p*-azidophenylalanine
BioID: proximity-dependent biotin labeling
CAT: chloramphenicol
co-IP: co-immunoprecipitation
DDA: data-dependent acquisition
DHFR-TS: dihydrofolate reductase-thymidylate synthase
DMEM: Dulbecco’s Modified Eagle Medium
FUDR: 5-fluorodeoxyuridine
GPI: glycosylphosphatidylinositol
HA: hemagglutinin
HDR: homology directed repair
HFF: human foreskin fibroblast
HXGPRT: hypoxanthine-xanthine-guanine phosphoribosyl transferase
ILP1: IMC localizing protein 1
IMC: inner membrane complex
IP: immunoprecipitation
LC-MS/MS: liquid chromatography tandem mass spectrometry
LFQ: label-free intensity-based quantitation
mAb: monoclonal antibody
MS/MS: tandem mass spectrometry
pAb: polyclonal antibody
PDB: Protein Data Bank
PSM: peptide-to-spectrum match
RIPA: radioimmunoprecipitation assay
SAG1: surface antigen 1
SDS: sodium dodecyl sulfate
UAA: unnatural amino acid
4Cys: quadruple C95S, C96S, C273S, C274S mutant

## References

[1] Hill DE, Chirukandoth S, Dubey JP. Biology and epidemiology of Toxoplasma gondii in man and animals. Anim Health Res Rev. 2005;6(1):41–61. 16164008 10.1079/ahr2005100

[2] Mackintosh CL, Beeson JG, Marsh K. Clinical features and pathogenesis of severe malaria. Trends in parasitology. 2004;20(12):597–603. 10.1016/j.pt.2004.09.006 15522670

[3] Sow SO, Muhsen K, Nasrin D, Blackwelder WC, Wu Y, Farag TH, et al The Burden of Cryptosporidium Diarrheal Disease among Children < 24 Months of Age in Moderate/High Mortality Regions of Sub-Saharan Africa and South Asia, Utilizing Data from the Global Enteric Multicenter Study (GEMS). PLoS Negl Trop Dis. 2016;10(5):e0004729 10.1371/journal.pntd.0004729 27219054 PMC4878811

[4] Dubey JP. Review of Neospora caninum and neosporosis in animals. Korean J Parasitol. 2003;41(1):1–16. 10.3347/kjp.2003.41.1.1 12666725 PMC2717477

[5] Kivaria FM. Estimated direct economic costs associated with tick-borne diseases on cattle in Tanzania. Trop Anim Health Prod. 2006;38(4):291–9. 10.1007/s11250-006-4181-2 17137131

[6] Sharman PA, Smith NC, Wallach MG, Katrib M. Chasing the golden egg: vaccination against poultry coccidiosis. Parasite Immunol. 2010;32(8):590–8. 10.1111/j.1365-3024.2010.01209.x 20626814

[7] Harding CR, Meissner M. The inner membrane complex through development of Toxoplasma gondii and Plasmodium. Cell Microbiol. 2014;16(5):632–41. 10.1111/cmi.12285 24612102 PMC4286798

[8] Boucher LE, Bosch J. The apicomplexan glideosome and adhesins—Structures and function. J Struct Biol. 2015;190(2):93–114. 10.1016/j.jsb.2015.02.008 25764948 PMC4417069

[9] Blader IJ, Coleman BI, Chen CT, Gubbels MJ. Lytic Cycle of Toxoplasma gondii: 15 Years Later. Annu Rev Microbiol. 2015;69:463–85. 10.1146/annurev-micro-091014-104100 26332089 PMC4659696

[10] Morrissette NS, Sibley LD. Cytoskeleton of apicomplexan parasites. Microbiol Mol Biol Rev. 2002;66(1):21–38; table of contents. 10.1128/MMBR.66.1.21-38.2002 11875126 PMC120781

[11] Frenal K, Polonais V, Marq JB, Stratmann R, Limenitakis J, Soldati-Favre D. Functional dissection of the apicomplexan glideosome molecular architecture. Cell Host Microbe. 2010;8(4):343–57. 10.1016/j.chom.2010.09.002 20951968

[12] Heaslip AT, Dzierszinski F, Stein B, Hu K. TgMORN1 is a key organizer for the basal complex of Toxoplasma gondii. PLoS Pathog. 2010;6(2):e1000754 10.1371/journal.ppat.1000754 20140195 PMC2816694

[13] Chen AL, Kim EW, Toh JY, Vashisht AA, Rashoff AQ, Van C, et al Novel components of the Toxoplasma inner membrane complex revealed by BioID. MBio. 2015;6(1):e02357–14. 10.1128/mBio.02357-14 25691595 PMC4337574

[14] Chen AL, Moon AS, Bell HN, Huang AS, Vashisht AA, Toh JY, et al Novel insights into the composition and function of the Toxoplasma IMC sutures. Cell Microbiol. 2016. 10.1111/cmi.12678 PMC5909696 27696623

[15] Anderson-White BR, Ivey FD, Cheng K, Szatanek T, Lorestani A, Beckers CJ, et al A family of intermediate filament-like proteins is sequentially assembled into the cytoskeleton of Toxoplasma gondii. Cell Microbiol. 2011;13(1):18–31. 10.1111/j.1462-5822.2010.01514.x 20698859 PMC3005026

[16] Gould SB, Tham WH, Cowman AF, McFadden GI, Waller RF. Alveolins, a new family of cortical proteins that define the protist infrakingdom Alveolata. Mol Biol Evol. 2008;25(6):1219–30. 10.1093/molbev/msn070 18359944

[17] Lorestani A, Ivey FD, Thirugnanam S, Busby MA, Marth GT, Cheeseman IM, et al Targeted proteomic dissection of Toxoplasma cytoskeleton sub-compartments using MORN1. Cytoskeleton (Hoboken). 2012;69(12):1069–85. 23027733 10.1002/cm.21077 PMC3566231

[18] Tremp AZ, Carter V, Saeed S, Dessens JT. Morphogenesis of Plasmodium zoites is uncoupled from tensile strength. Mol Microbiol. 2013;89(3):552–64. 10.1111/mmi.12297 23773015 PMC3912903

[19] Wang L. Engineering the Genetic Code in Cells and Animals: Biological Considerations and Impacts. Acc Chem Res. 2017;50(11):2767–75. 10.1021/acs.accounts.7b00376 28984438 PMC5698093

[20] Wang L, Xie J, Schultz PG. Expanding the Genetic Code. Annual Review of Biophysics and Biomolecular Structure. 2006;35(1):225–49. 10.1146/annurev.biophys.35.101105.121507 16689635

[21] Chin JW, Cropp TA, Anderson JC, Mukherji M, Zhang Z, Schultz PG. An Expanded Eukaryotic Genetic Code. *Science*. 2003;301(5635):964–7. 10.1126/science.1084772 12920298

[22] Takimoto JK, Adams KL, Xiang Z, Wang L. Improving orthogonal tRNA-synthetase recognition for efficient unnatural amino acid incorporation and application in mammalian cells. Mol Biosyst. 2009;5(9):931–4. 10.1039/b904228h 19668857

[23] Coin I, Katritch V, Sun T, Xiang Z, Siu FY, Beyermann M, et al Genetically encoded chemical probes in cells reveal the binding path of urocortin-I to CRF class B GPCR. Cell. 2013;155(6):1258–69. 10.1016/j.cell.2013.11.008 24290358 PMC3916339

[24] Sidik SM, Hackett CG, Tran F, Westwood NJ, Lourido S. Efficient genome engineering of Toxoplasma gondii using CRISPR/Cas9. PLoS ONE. 2014;9(6):e100450 10.1371/journal.pone.0100450 24971596 PMC4074098

[25] Burg JL, Perelman D, Kasper LH, Ware PL, Boothroyd JC. Molecular analysis of the gene encoding the major surface antigen of Toxoplasma gondii. J Immunol. 1988;141(10):3584–91. 3183382

[26] Kim K, Boothroyd JC. Toxoplasma gondii: stable complementation of sag1 (p30) mutants using SAG1 transfection and fluorescence-activated cell sorting. Exp Parasitol. 1995;80(1):46–53. 10.1006/expr.1995.1006 7821410

[27] Sakamoto K, Hayashi A, Sakamoto A, Kiga D, Nakayama H, Soma A, et al Site-specific incorporation of an unnatural amino acid into proteins in mammalian cells. Nucleic Acids Res. 2002;30(21):4692–9. 10.1093/nar/gkf589 12409460 PMC135798

[28] Schumacher MA, Bashor CJ, Song MH, Otsu K, Zhu S, Parry RJ, et al The structural mechanism of GTP stabilized oligomerization and catalytic activation of the Toxoplasma gondii uracil phosphoribosyltransferase. Proc Natl Acad Sci U S A. 2002;99(1):78–83. 10.1073/pnas.012399599 11773618 PMC117517

[29] Schumacher MA, Carter D, Scott DM, Roos DS, Ullman B, Brennan RG. Crystal structures of Toxoplasma gondii uracil phosphoribosyltransferase reveal the atomic basis of pyrimidine discrimination and prodrug binding. EMBO J. 1998;17(12):3219–32. 10.1093/emboj/17.12.3219 9628859 PMC1170660

[30] Ren J, Wen L, Gao X, Jin C, Xue Y, Yao X. CSS-Palm 2.0: an updated software for palmitoylation sites prediction. Protein Eng Des Sel. 2008;21(11):639–44. 10.1093/protein/gzn039 18753194 PMC2569006

[31] Kelley LA, Mezulis S, Yates CM, Wass MN, Sternberg MJ. The Phyre2 web portal for protein modeling, prediction and analysis. Nat Protoc. 2015;10(6):845–58. 10.1038/nprot.2015.053 25950237 PMC5298202

[32] Lupas A. Predicting coiled-coil regions in proteins. Curr Opin Struct Biol. 1997;7(3):388–93. 9204281 10.1016/s0959-440x(97)80056-5

[33] Lupas AN, Bassler J. Coiled Coils—A Model System for the 21st Century. Trends Biochem Sci. 2017;42(2):130–40. 10.1016/j.tibs.2016.10.007 27884598

[34] Drozdetskiy A, Cole C, Procter J, Barton GJ. JPred4: a protein secondary structure prediction server. Nucleic Acids Res. 2015;43(W1):W389–94. 10.1093/nar/gkv332 25883141 PMC4489285

[35] Dubey R, Harrison B, Dangoudoubiyam S, Bandini G, Cheng K, Kosber A, et al Differential Roles for Inner Membrane Complex Proteins across Toxoplasma gondii and Sarcocystis neurona Development. mSphere. 2017;2(5). 10.1128/mSphere.00409-17 PMC5646244 29062899

[36] Wals K, Ovaa H. Unnatural amino acid incorporation in E. coli: current and future applications in the design of therapeutic proteins. Front Chem. 2014;2:15 10.3389/fchem.2014.00015 24790983 PMC3982533

[37] Nakamura Y, Gojobori T, Ikemura T. Codon usage tabulated from international DNA sequence databases: status for the year 2000. Nucleic Acids Res. 2000;28(1):292 10.1093/nar/28.1.292 10592250 PMC102460

[38] Wier GM, McGreevy EM, Brown MJ, Boyle JP. New method for the orthogonal labeling and purification of Toxoplasma gondii proteins while inside the host cell. MBio. 2015;6(2):e01628 10.1128/mBio.01628-14 25759504 PMC4453564

[39] Mehnert M, Sommer T, Jarosch E. Der1 promotes movement of misfolded proteins through the endoplasmic reticulum membrane. Nat Cell Biol. 2014;16(1):77–86. 10.1038/ncb2882 24292014

[40] Mueller-Planitz F. Crossfinder-assisted mapping of protein crosslinks formed by site-specifically incorporated crosslinkers. Bioinformatics. 2015;31(12):2043–5. 10.1093/bioinformatics/btv083 25788624

[41] Sidik SM, Huet D, Ganesan SM, Huynh MH, Wang T, Nasamu AS, et al A Genome-wide CRISPR Screen in Toxoplasma Identifies Essential Apicomplexan Genes. Cell. 2016;166(6):1423–35 e12. 10.1016/j.cell.2016.08.019 27594426 PMC5017925

[42] Volkmann K, Pfander C, Burstroem C, Ahras M, Goulding D, Rayner JC, et al The alveolin IMC1h is required for normal ookinete and sporozoite motility behaviour and host colonisation in Plasmodium berghei. PLoS ONE. 2012;7(7):e41409 10.1371/journal.pone.0041409 22844474 PMC3402405

[43] Zhang M, Wang C, Otto TD, Oberstaller J, Liao X, Adapa SR, et al Uncovering the essential genes of the human malaria parasite Plasmodium falciparum by saturation mutagenesis. Science. 2018;360(6388). 10.1126/science.aap7847 PMC6360947 29724925

[44] Long S, Anthony B, Drewry LL, Sibley LD. A conserved ankyrin repeat-containing protein regulates conoid stability, motility and cell invasion in Toxoplasma gondii. Nat Commun. 2017;8(1):2236 10.1038/s41467-017-02341-2 29269729 PMC5740107

[45] Benchimol M. Trichomonads under Microscopy. Microsc Microanal. 2004;10(5):528–50. 10.1017/S1431927604040905 15525428

[46] Brown JR, Schwartz CL, Heumann JM, Dawson SC, Hoenger A. A detailed look at the cytoskeletal architecture of the Giardia lamblia ventral disc. J Struct Biol. 2016;194(1):38–48. 10.1016/j.jsb.2016.01.011 26821343 PMC4764415

[47] Nosala C, Hagen KD, Dawson SC. 'Disc-o-Fever': Getting Down with Giardia's Groovy Microtubule Organelle. Trends Cell Biol. 2018;28(2):99–112. 10.1016/j.tcb.2017.10.007 29153830 PMC7864154

[48] Perdomo D, Bonhivers M, Robinson DR. The Trypanosome Flagellar Pocket Collar and Its Ring Forming Protein-TbBILBO1. Cells. 2016;5(1). 10.3390/cells5010009 PMC4810094 26950156

[49] Vaughan S, Gull K. Basal body structure and cell cycle-dependent biogenesis in Trypanosoma brucei. Cilia. 2015;5:5 10.1186/s13630-016-0023-7 26862392 PMC4746817

[50] Donald RG, Carter D, Ullman B, Roos DS. Insertional tagging, cloning, and expression of the Toxoplasma gondii hypoxanthine-xanthine-guanine phosphoribosyltransferase gene. Use as a selectable marker for stable transformation. J Biol Chem. 1996;271(24):14010–9. 10.1074/jbc.271.24.14010 8662859

[51] Donald RG, Roos DS. Stable molecular transformation of Toxoplasma gondii: a selectable dihydrofolate reductase-thymidylate synthase marker based on drug-resistance mutations in malaria. Proc Natl Acad Sci U S A. 1993;90(24):11703–7. 10.1073/pnas.90.24.11703 8265612 PMC48052

[52] Kim K, Soldati D, Boothroyd JC. Gene replacement in Toxoplasma gondii with chloramphenicol acetyltransferase as selectable marker. Science. 1993;262(5135):911–4. 10.1126/science.8235614 8235614

[53] Donald RG, Roos DS. Insertional mutagenesis and marker rescue in a protozoan parasite: cloning of the uracil phosphoribosyltransferase locus from Toxoplasma gondii. Proc Natl Acad Sci U S A. 1995;92(12):5749–53. 10.1073/pnas.92.12.5749 7777580 PMC41774

[54] Saeij JP, Boyle JP, Coller S, Taylor S, Sibley LD, Brooke-Powell ET, et al Polymorphic secreted kinases are key virulence factors in toxoplasmosis. Science. 2006;314(5806):1780–3. 10.1126/science.1133690 17170306 PMC2646183

[55] Huynh MH, Carruthers VB. Tagging of endogenous genes in a Toxoplasma gondii strain lacking Ku80. Eukaryot Cell. 2009;8(4):530–9. 10.1128/EC.00358-08 19218426 PMC2669203

[56] Konrad C, Wek RC, Sullivan WJ Jr. A GCN2-like eukaryotic initiation factor 2 kinase increases the viability of extracellular Toxoplasma gondii parasites. Eukaryot Cell. 2011;10(11):1403–12. 10.1128/EC.05117-11 21908594 PMC3209059

[57] Bastin P, Bagherzadeh Z, Matthews KR, Gull K. A novel epitope tag system to study protein targeting and organelle biogenesis in Trypanosoma brucei. Mol Biochem Parasitol. 1996;77(2):235–9. 10.1016/0166-6851(96)02598-4 8813669

[58] Evan GI, Lewis GK, Ramsay G, Bishop JM. Isolation of monoclonal antibodies specific for human c-myc proto-oncogene product. Mol Cell Biol. 1985;5(12):3610–6. 10.1128/mcb.5.12.3610 3915782 PMC369192

[59] Dunn JD, Ravindran S, Kim SK, Boothroyd JC. The Toxoplasma gondii dense granule protein GRA7 is phosphorylated upon invasion and forms an unexpected association with the rhoptry proteins ROP2 and ROP4. Infect Immun. 2008;76(12):5853–61. 10.1128/IAI.01667-07 18809661 PMC2583583

[60] Wichroski MJ, Melton JA, Donahue CG, Tweten RK, Ward GE. Clostridium septicum alpha-toxin is active against the parasitic protozoan Toxoplasma gondii and targets members of the SAG family of glycosylphosphatidylinositol-anchored surface proteins. Infect Immun. 2002;70(8):4353–61. 10.1128/IAI.70.8.4353-4361.2002 12117945 PMC128134

[61] Beck JR, Rodriguez-Fernandez IA, de Leon JC, Huynh MH, Carruthers VB, Morrissette NS, et al A novel family of Toxoplasma IMC proteins displays a hierarchical organization and functions in coordinating parasite division. PLoS Pathog. 2010;6(9):e1001094 10.1371/journal.ppat.1001094 20844581 PMC2936552

[62] Rome ME, Beck JR, Turetzky JM, Webster P, Bradley PJ. Intervacuolar transport and unique topology of GRA14, a novel dense granule protein in Toxoplasma gondii. Infect Immun. 2008;76(11):4865–75. 10.1128/IAI.00782-08 18765740 PMC2573327

[63] Bradley PJ, Ward C, Cheng SJ, Alexander DL, Coller S, Coombs GH, et al Proteomic analysis of rhoptry organelles reveals many novel constituents for host-parasite interactions in Toxoplasma gondii. J Biol Chem. 2005;280(40):34245–58. 10.1074/jbc.M504158200 16002398

[64] Reese ML, Zeiner GM, Saeij JP, Boothroyd JC, Boyle JP. Polymorphic family of injected pseudokinases is paramount in Toxoplasma virulence. Proc Natl Acad Sci U S A. 2011;108(23):9625–30. 10.1073/pnas.1015980108 21436047 PMC3111280

[65] Nadipuram SM, Kim EW, Vashisht AA, Lin AH, Bell HN, Coppens I, et al In Vivo Biotinylation of the Toxoplasma Parasitophorous Vacuole Reveals Novel Dense Granule Proteins Important for Parasite Growth and Pathogenesis. MBio. 2016;7(4). 10.1128/mBio.00808-16 PMC4981711 27486190

[66] Cox J, Mann M. MaxQuant enables high peptide identification rates, individualized p.p.b.-range mass accuracies and proteome-wide protein quantification. Nat Biotechnol. 2008;26(12):1367–72. 10.1038/nbt.1511 19029910

[67] Madeira F, Park YM, Lee J, Buso N, Gur T, Madhusoodanan N, et al The EMBL-EBI search and sequence analysis tools APIs in 2019. Nucleic Acids Res. 2019. 10.1093/nar/gkz268 PMC6602479 30976793

