# Supplementary material for: A photoactivatable crosslinking system reveals protein interactions in the Toxoplasma gondii inner membrane complex
Source: PLoS Biol. 2019 Oct 4;17(10):e3000475. doi: 10.1371/journal.pbio.3000475 (PMC6795473; doi:10.1371/journal.pbio.3000475)
Supplement: S1 Fig — ILP1 is well conserved in coccidians, while the Plasmodium falciparum ortholog is more divergent. The likely myristoylated glycine at position 2 is conserved. Two putatively palmitoylated cysteine pairs are present in the Toxoplasma sequence at residues C95, C96 and C273, C274. The internal pair is present in Plasmodium but missing in Eimeria, while the C-terminal pair is conserved in coccidians but only present as a single cysteine in Plasmodium. Alignment was generated using Clustal Omega [67] and shaded using BoxShade (https://embnet.vital-it.ch/software/BOX_form.html). Black highlights represent identity; gray highlights represent similarity. ILP1, IMC localizing protein 1 (DOCX) [file pbio.3000475.s001.docx]

TgME49 1 MGQKLTVEERRNIKREVYMIYPGLEQQLEMAFTCHDLQHEGKLPYTTLEPIIRHLLMQYG
NcLIV 1 MGQKLTVEERRNIKREVYMLYPGLEQQLEMAFTCHDLQHEGKLPYTTLEPIIRHLLMQYG
EtHoughton 1 MGQSLTTQEKHNIKREIYTLYPGLEQQLEMAFSCHDIEGTLTLPYATLEPVIRHLLMQYG
Pf3D7 1 MGQIS-SKEDEIEKQNIYATYPGLEQQLDMVFACHDISKQGKLSYKTVEMILRHFLMQCG


TgME49 61 LIEYVTRFSDSEGCLDANQIRAELNEFGVNTG-GLCCGSSLTLEDFKSLAVIWLRKILDC
NcLIV 61 LIEYVTRFSDSAGCLDANQVRAELNEFNVNTS-GLCCGSSLTLEDFKSLAVIWLRKILDS
EtHoughton 61 LIEYVTRFSKESGALDPSHVRHELAEFGVATT-RLFGDANLNVDDFKSLAVVWLKKILDT
Pf3D7 60 FMEYVCRFVDENGTLDLKHVSNYLSIKKLMYKLKCCGESMLTLDEMKELVIIFLKKISDT


TgME49 120 HADDQAVWMAKLKAEQEEQAEAYTRAMREFQDTFTQQHALYQQGLQEQQKQINDWNKLLE
NcLIV 120 HADDQAVWMSKLKAEQEEQAEAYTRAMREFQETFTQQHALYQQGLQEQQKQISDWNKLLE
EtHoughton 120 HADDQAEWMEKLRADQEEQSANYAKAMKEFQEQYVKQQAVYQQGLEEQQKQIQDWNRLLE
Pf3D7 120 YTEDQTKWLEQMKSSQEQQDKALEEAMYKYEKNILFHHAVKEQQILQNDKKLNEWNENVE


TgME49 180 DAQKTQQTIYEQEVRRMEEARLKEATAAEEAMKEQIDLISQYKEKLEKIAA--ADTSGKC
NcLIV 180 DAQKTQQHIYEQEMRRMEEARQKEASAAEEAMKEQMDLISQYKEKLEKIAA--ADKSGKC
EtHoughton 180 DAHKTQQQIYDEEVRRMKEIQEREARASEIARQEELELIRKYQQKLEEIAR---SEGGKC
Pf3D7 180 NAYEAQQEILRQFE----SSRKKNI-DISLEKNNELIIAKDYIDKIKEAATDNKYDNSKC


TgME49 238 FVYPAAATPYGACASAGAQEPTRRRRVK---KEHPSRACC
NcLIV 238 FVYPAAATPYGACASAGAQEPTRRRRVK---KEHQSRACC
EtHoughton 237 FVYPASKTPYGACASAGAQEPCRRKRSQARREQRPAKGCC
Pf3D7 235 FIYPASSAPCGACTSAGAIIHHRRYKEKRRKKEY--SLCL
