# Supplementary figures and images for: A photoactivatable crosslinking system reveals protein interactions in the Toxoplasma gondii inner membrane complex
Source: PLoS Biol. 2019 Oct 4;17(10):e3000475. doi: 10.1371/journal.pbio.3000475 (PMC6795473; doi:10.1371/journal.pbio.3000475)

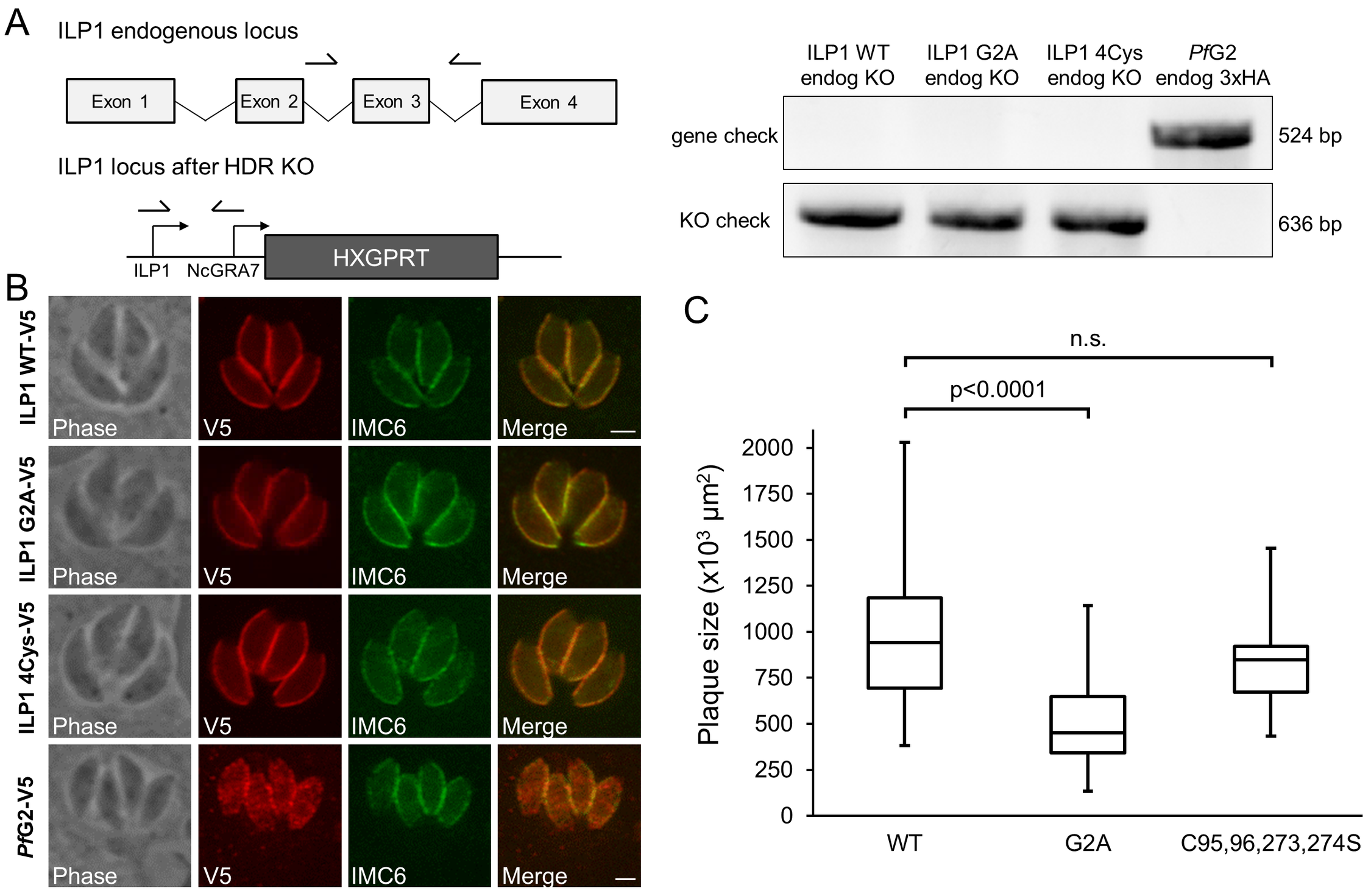

Supplement: S2 Fig — Loss-of-function mutations of the putative myristoylation (G2A) and palmitoylation (4Cys) sites were assessed by knocking in mutant copies to the UPRT locus and disrupting the endogenous ILP1 locus using CRISPR/Cas9. (A) Strategy and PCR analysis of endogenous ILP1 disruption. Knockouts were assessed by absence of amplification using intronic primers and positive amplification of the sequence between the ILP1 promoter and the NcGra7 promoter following HDR. (B) IFA showing that exogenous ILP1 WT, G2A, and 4Cys copies all localize normally to the parasite periphery. The Plasmodium G2 ortholog fails to localize properly and could not compensate for the endogenous ILP1 knockout. Red, mouse anti-V5 antibody; green, rabbit anti-IMC6 antibody. Scale bars represent 2 μm. (C) Plaque assays of the ILP1 mutants following knockout of endogenous ILP1. The ILP1 G2A mutant has a slight but significant growth defect when compared with the ILP1 WT strain (an approximate 50% reduction). The 4Cys mutant does not have any growth disadvantage compared with control. HDR, homology directed repair; IFA, immunofluorescence assay; ILP1, IMC localizing protein 1; IMC, inner membrane complex; NcGra7, Neospora caninum Gra7 promoter; UPRT, uracil phosphoribosyltransferase; WT, wild-type; 4Cys, quadruple C95S, C96S, C273S, C274S mutant (TIF) [file pbio.3000475.s002.tif]

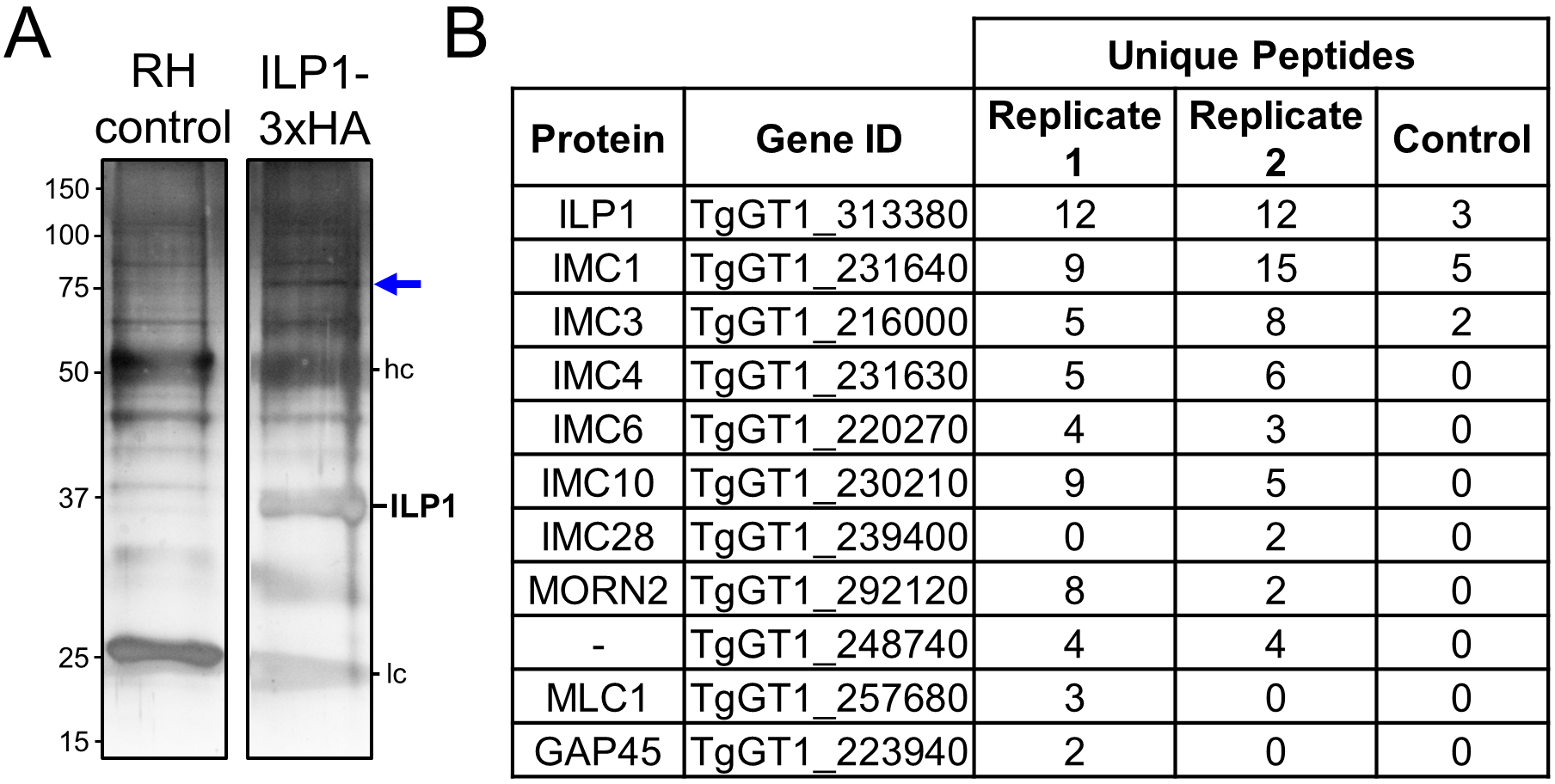

Supplement: S3 Fig — (A) Representative silver stain of an anti-HA IP of ILP1-3xHA parasites performed after fractionation in 1% Triton-X 100 and extensive sonication of the pellet to solubilize the IMC cytoskeleton. RH parasites were used as a control. A gel slice containing a unique band (blue arrow) was excised and proteins were identified by mass spectrometry. Identified proteins included the alveolins and components of the glideosome. co-IP, co-immunoprecipitation; HA, hemagglutinin; ILP1, IMC localizing protein 1; IMC, inner membrane complex (TIF) [file pbio.3000475.s003.tif]
