# Supplementary material for: A photoactivatable crosslinking system reveals protein interactions in the Toxoplasma gondii inner membrane complex
Source: PLoS Biol. 2019 Oct 4;17(10):e3000475. doi: 10.1371/journal.pbio.3000475 (PMC6795473; doi:10.1371/journal.pbio.3000475)
Supplement: S1 Text — (DOCX) [file pbio.3000475.s006.docx]

**S6 Text: List of gene fragments used in this study (5’ to 3’).**

Partial Toxoplasma U6 promoter, BstYam tRNA cassette (AvrII, XbaI flanks)

CCTAGGCCTGACGCGCCTCCTGCAGAACGCGAGACACTGGGATATGTAGAGCCAAGGGGGAAACCTTCGAACTCTCGAATGTCTTCTCTGACAAGAATCATATTTCCATCAGTTCTGTCAGATTTTCAAATGGCGACCTGCAGAGGCCTGCTTCCTCCCTGTGCGCTCTTCGAAGGGGCTTTCTGTCGCGCAGGGTCACCTCGTCCCCGAAGGGGGTGTTTGCCTTCTGGTAAATGGGGATGTCAAGTTGGAGGGGTAGCGAAGTGGCTAAACGCGGCGGACTCTAAATCCGCTCCCTTTGGGTTCGGCGGTTCGAATCCGTCCCCCTCCATTTTTTTTTCTTTTTCtctaga

*Plasmodium* G2 coding sequence codon optimized for *Toxoplasma*

ATGGGCCAGATCAGTTCCAAGGAAGATGAGATTGAAAAGCAAAATATCTATGCGACTTACCCTGGACTGGAACAGCAGCTTGATATGGTGTTCGCGTGCCATGACATTTCTAAACAAGGAAAGTTGTCGTACAAGACAGTTGAAATGATCCTGAGGCACTTTTTGATGCAATGCGGGTTTATGGAGTACGTGTGCAGATTTGTCGACGAGAACGGAACCTTGGATTTGAAGCACGTGTCCAATTATCTTTCTATCAAGAAATTGATGTACAAATTGAAATGCTGCGGGGAAAGTATGCTCACGCTGGACGAAATGAAAGAGTTGGTTATTATCTTCCTCAAGAAAATTTCCGACACCTATACCGAGGATCAGACCAAATGGCTGGAGCAAATGAAGTCGTCGCAAGAGCAACAAGATAAGGCTCTCGAAGAGGCTATGTATAAATACGAAAAGAACATTCTGTTTCATCATGCGGTGAAGGAACAGCAAATTCTTCAGAATGATAAAAAACTGAATGAGTGGAATGAGAACGTGGAGAATGCGTATGAGGCACAACAAGAAATTTTGCGGCAATTCGAATCCTCCAGGAAGAAAAATATTGATATTTCTCTGGAGAAAAACAACGAACTGATTATCGCAAAAGACTATATTGACAAGATCAAGGAAGCTGCTACGGATAACAAGTATGATAATTCCAAATGTTTCATCTATCCAGCGTCTTCTGCGCCTTGCGGCGCATGTACGAGCGCTGGCGCCATCATCCACCATAGGCGATACAAGGAAAAACGTAGGAAGAAAGAGTATAGCTTGTGCTTA
