# Supplementary material for: A photoactivatable crosslinking system reveals protein interactions in the Toxoplasma gondii inner membrane complex
Source: PLoS Biol. 2019 Oct 4;17(10):e3000475. doi: 10.1371/journal.pbio.3000475 (PMC6795473; doi:10.1371/journal.pbio.3000475)
Supplement: S1 Raw images — (PDF) [file pbio.3000475.s007.pdf]

Figure 1

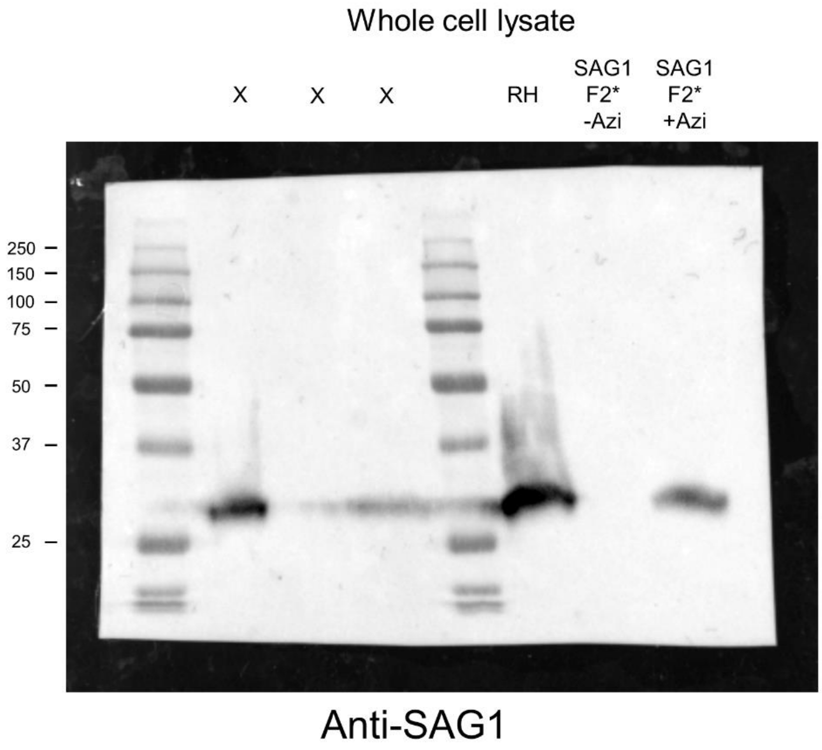
Whole cell lysate
X
X
X
RH
SAG1 F2* -Azi
SAG1 F2* +Azi
250
150
100
75
50
37
25
Anti-SAG1

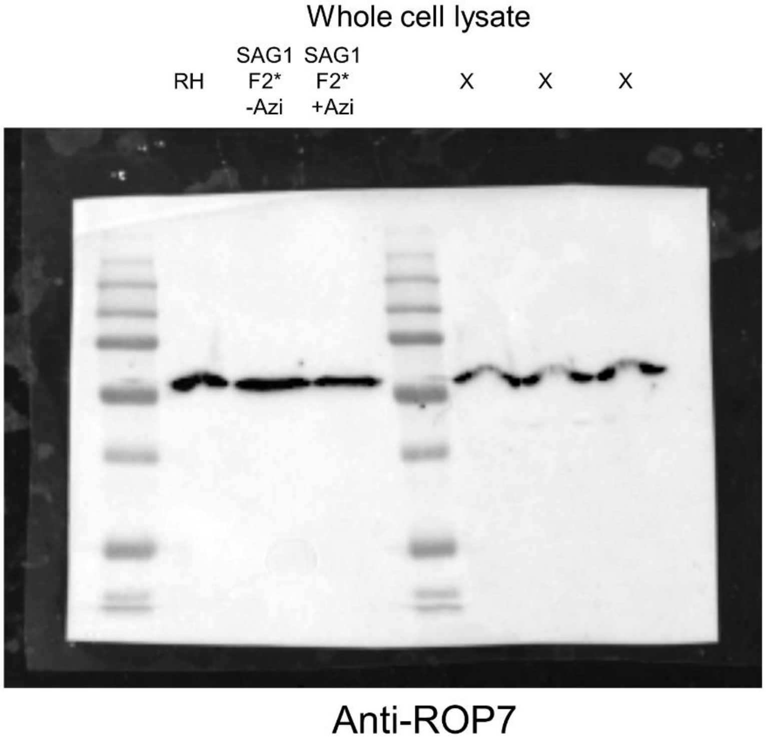
Whole cell lysate
RH
SAG1 F2* -Azi
SAG1 F2* +Azi
X
X
X
Anti-ROP7

Figure 2

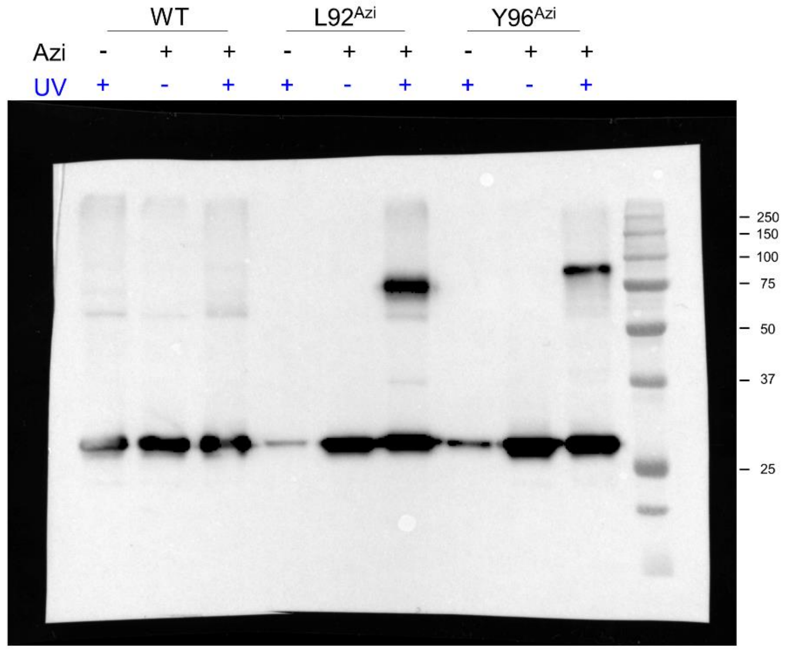

UPRT-HA blot

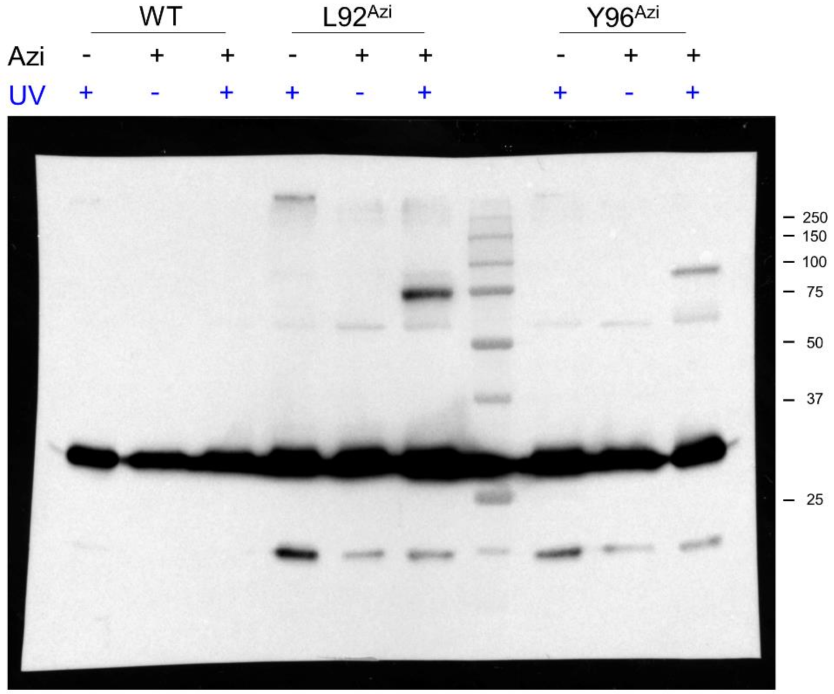

Myc-UPRT blot

Figure 3

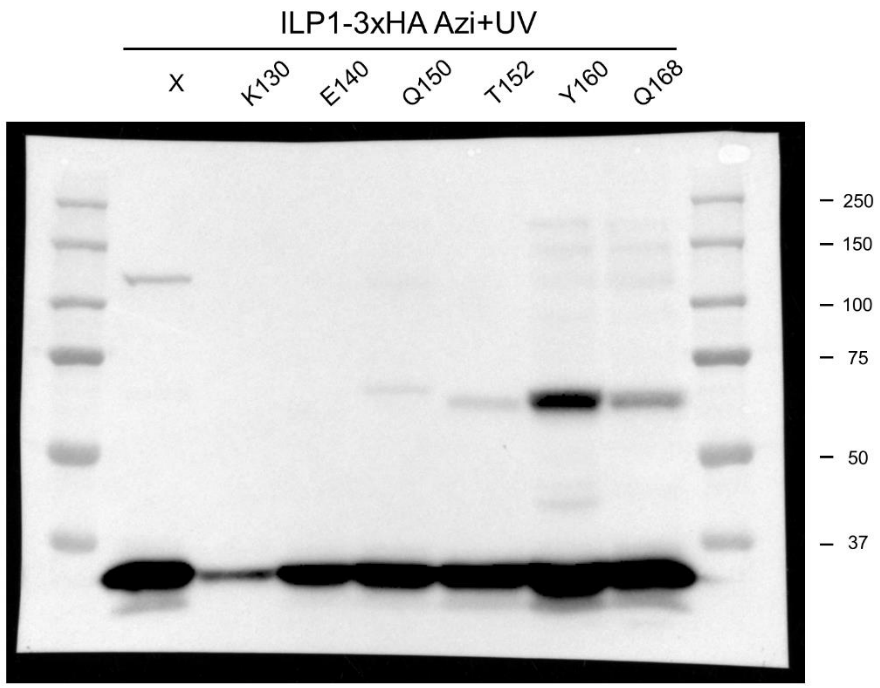

ILP1-HA blot

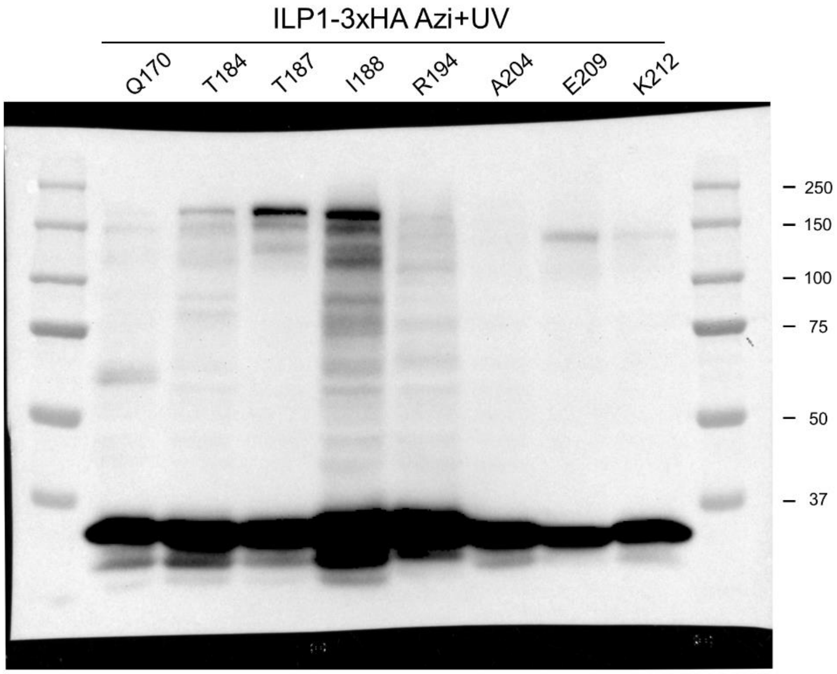

ILP1-HA blot

Figure 4

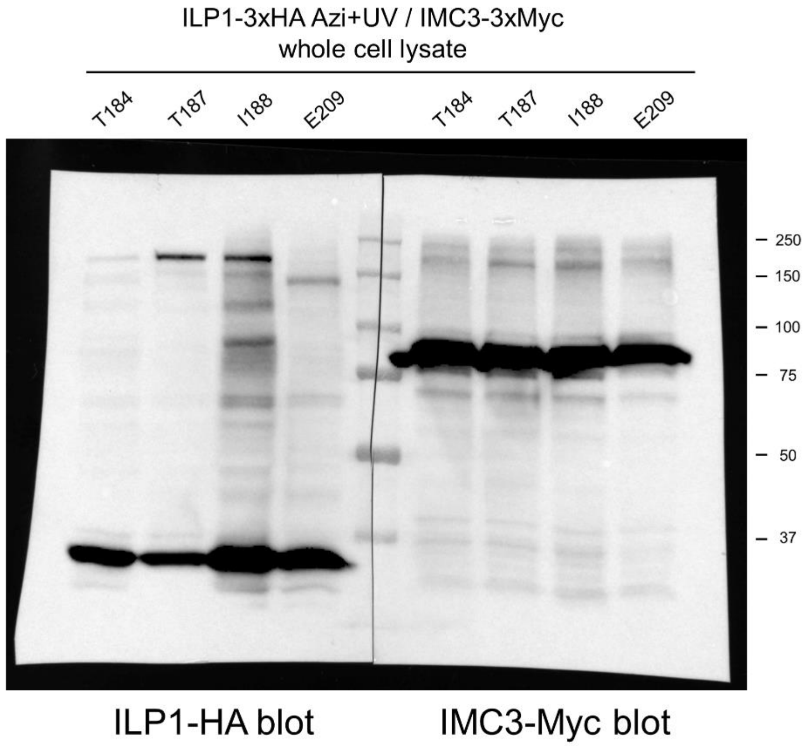

ILP1-3xHA Azi+UV / IMC3-3xMyc
whole cell lysate
T184
T187
I188
E209
T184
T187
I188
E209
250
150
100
75
50
37
ILP1-HA blot
IMC3-Myc blot

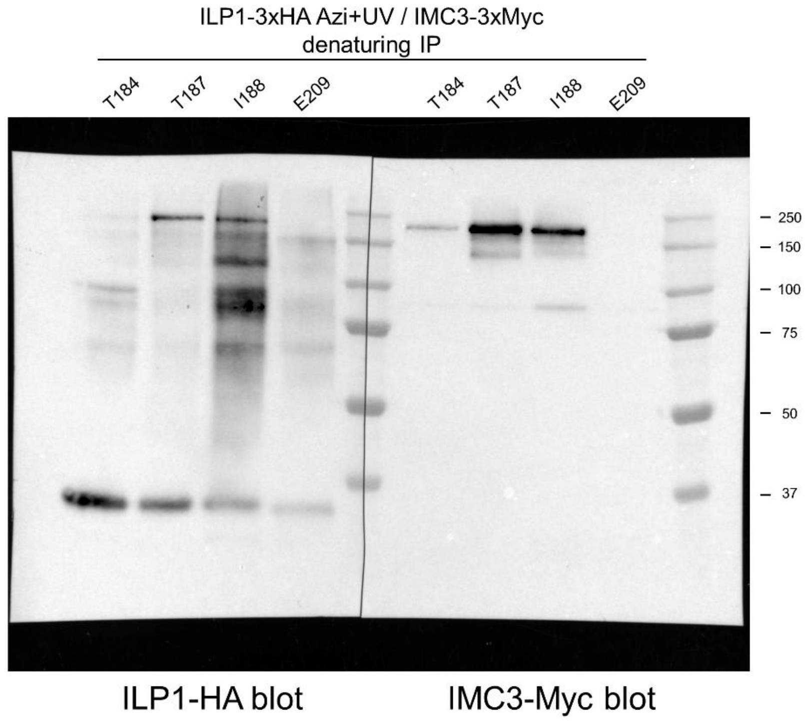

ILP1-3xHA Azi+UV / IMC3-3xMyc
denaturing IP
T184
T187
I188
E209
T184
T187
I188
E209
250
150
100
75
50
37
ILP1-HA blot
IMC3-Myc blot

Figure 5

Whole cell lysate

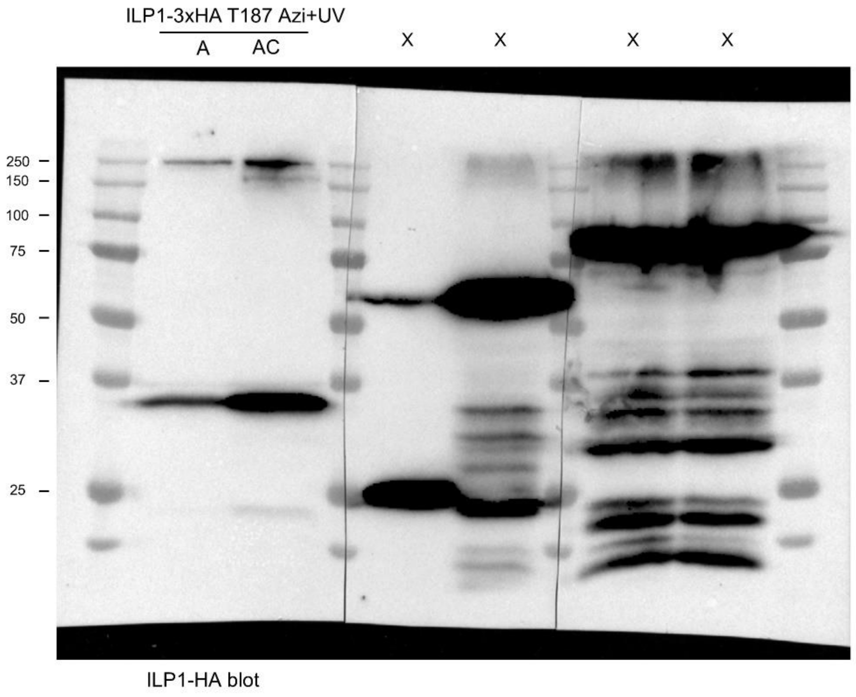

30 sec exposure anti-HA IP

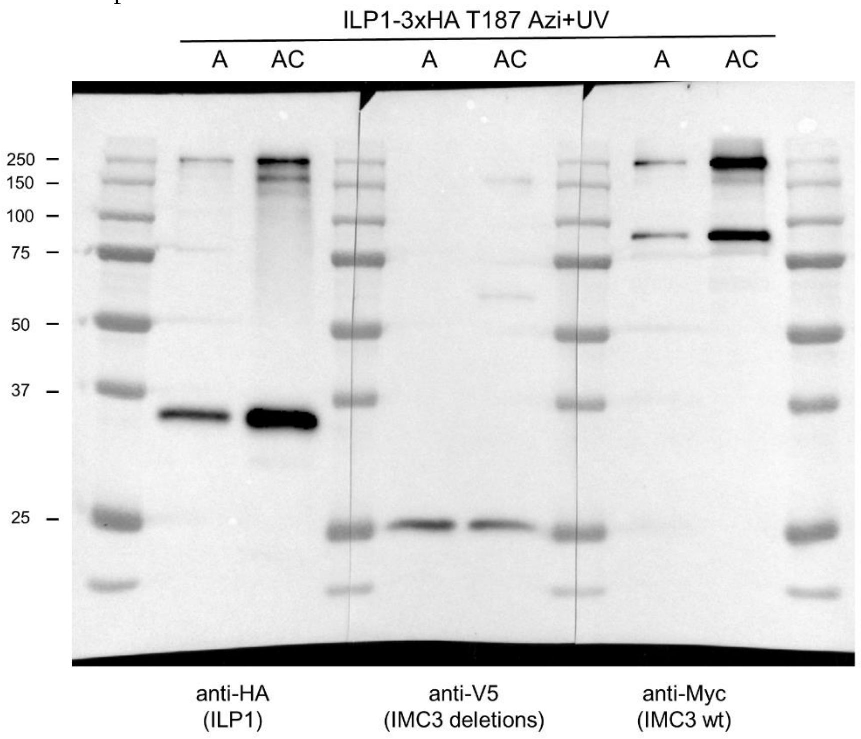

300 sec exposure anti-HA IP

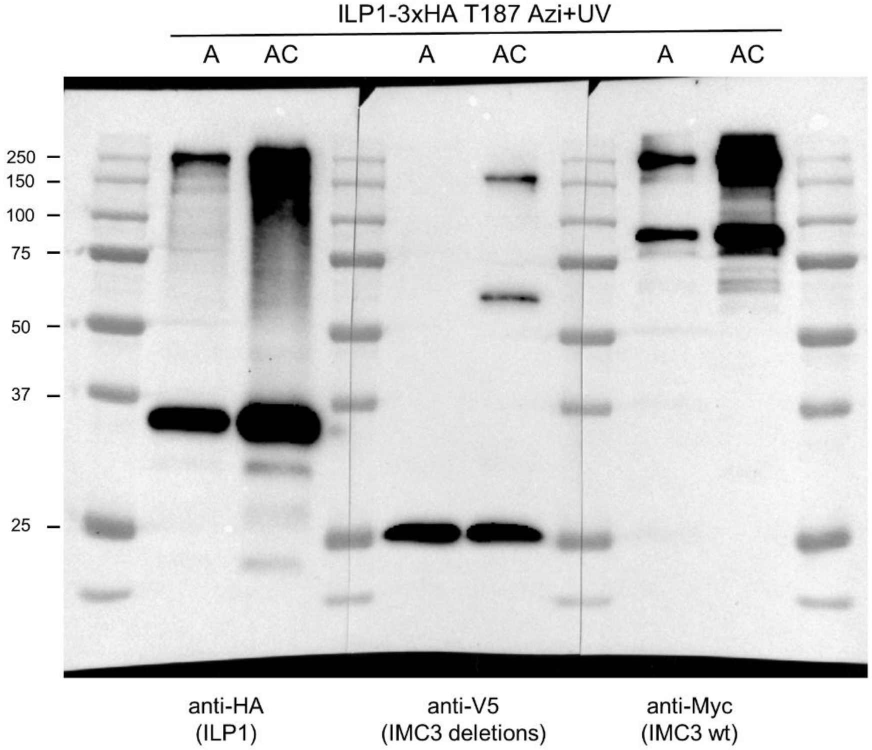

20 sec anti-V5 IP

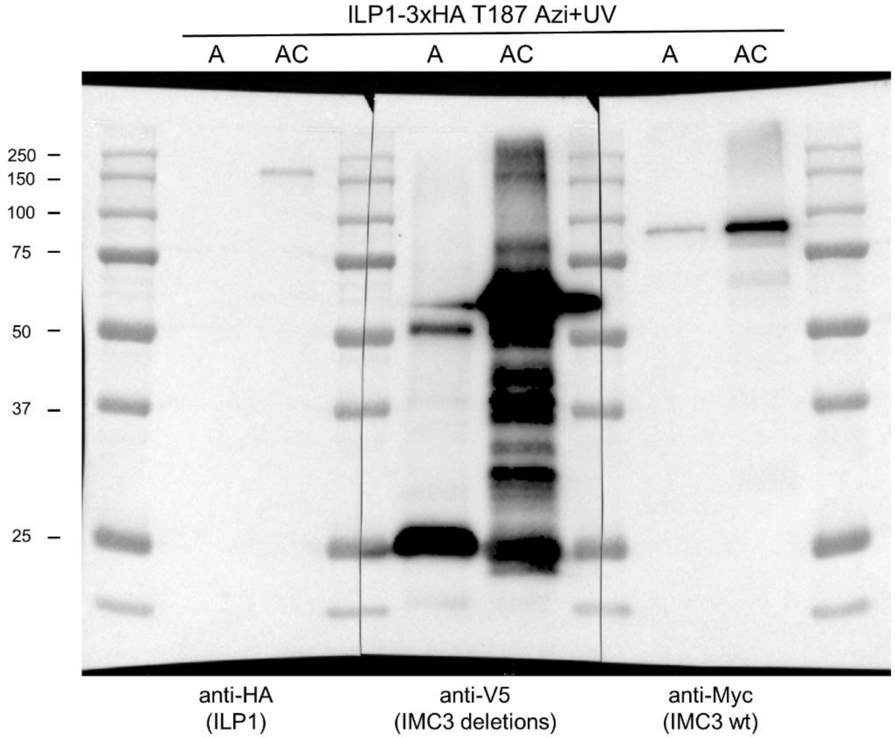

200 sec anti-V5 IP

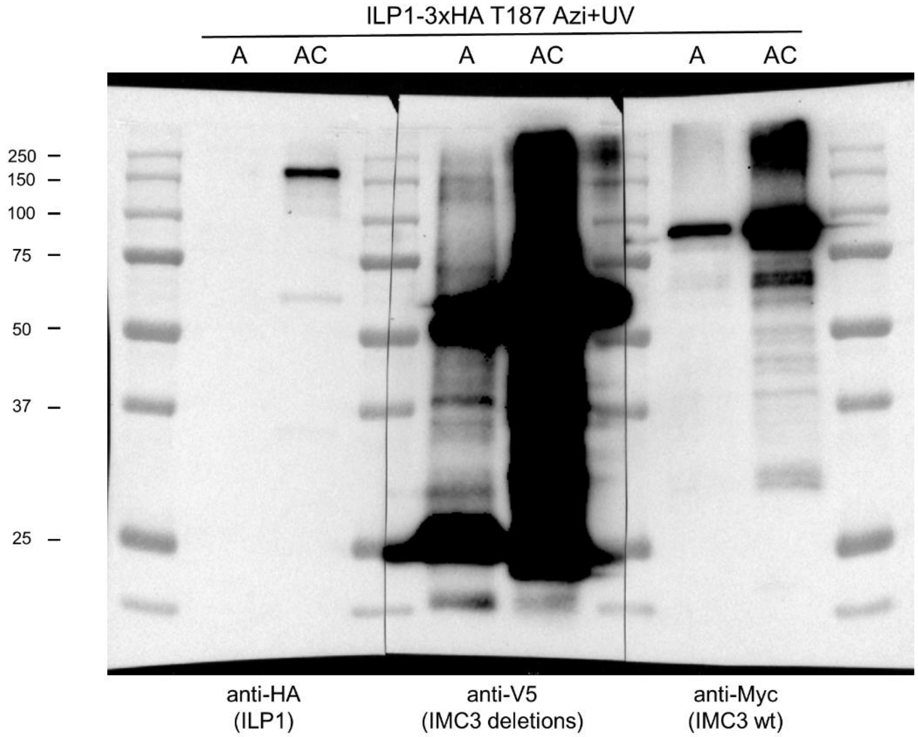

Figure 6

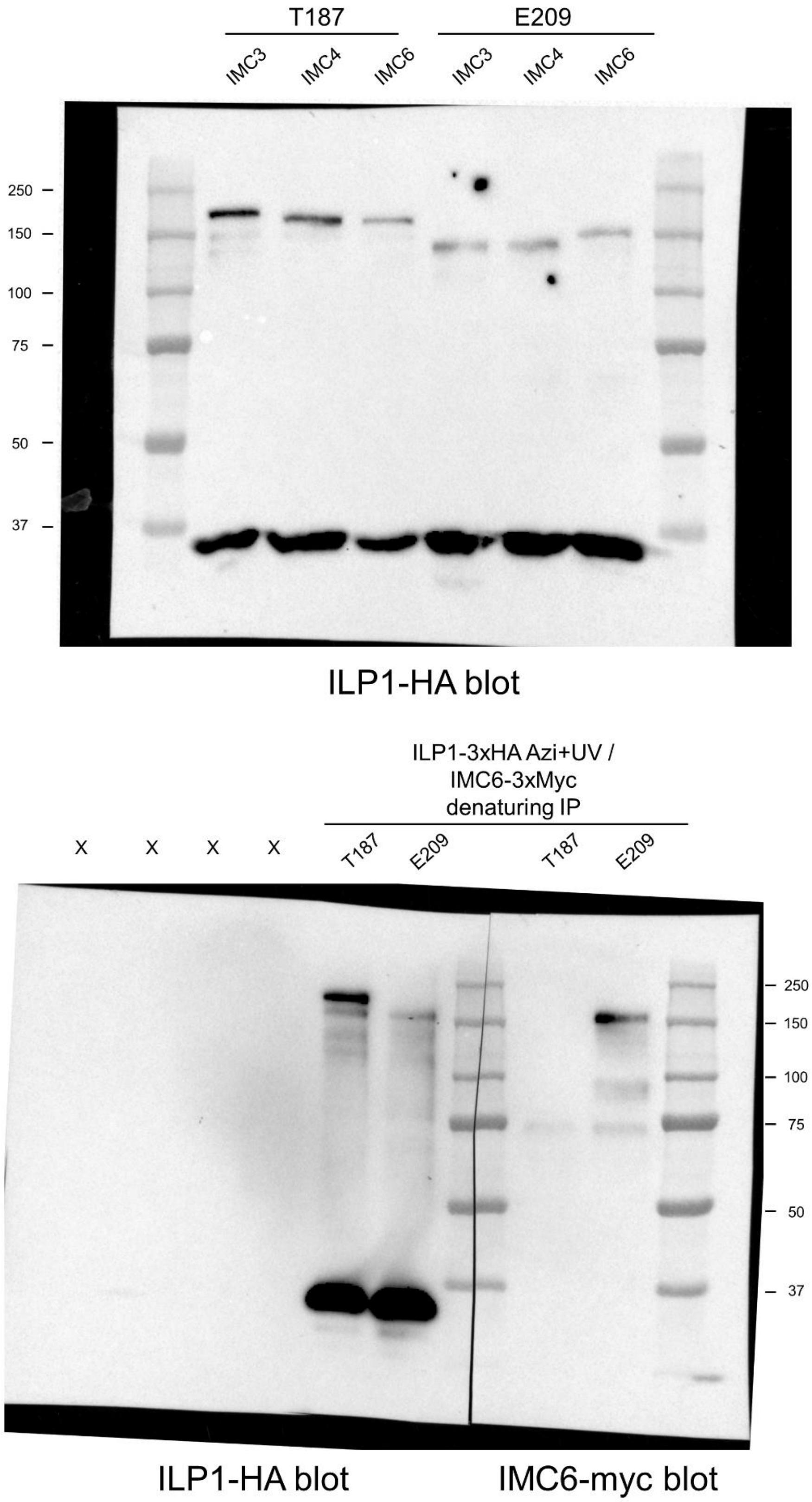
T187
E209
IMC3
IMC4
IMC6
IMC3
IMC4
IMC6
250
150
100
75
50
37
ILP1-HA blot
ILP1-3xHA Azi+UV /
IMC6-3xMyc
denaturing IP
X
X
X
X
T187
E209
T187
E209
250
150
100
75
50
37
ILP1-HA blot
IMC6-myc blot

Figure 7

Whole cell lysate

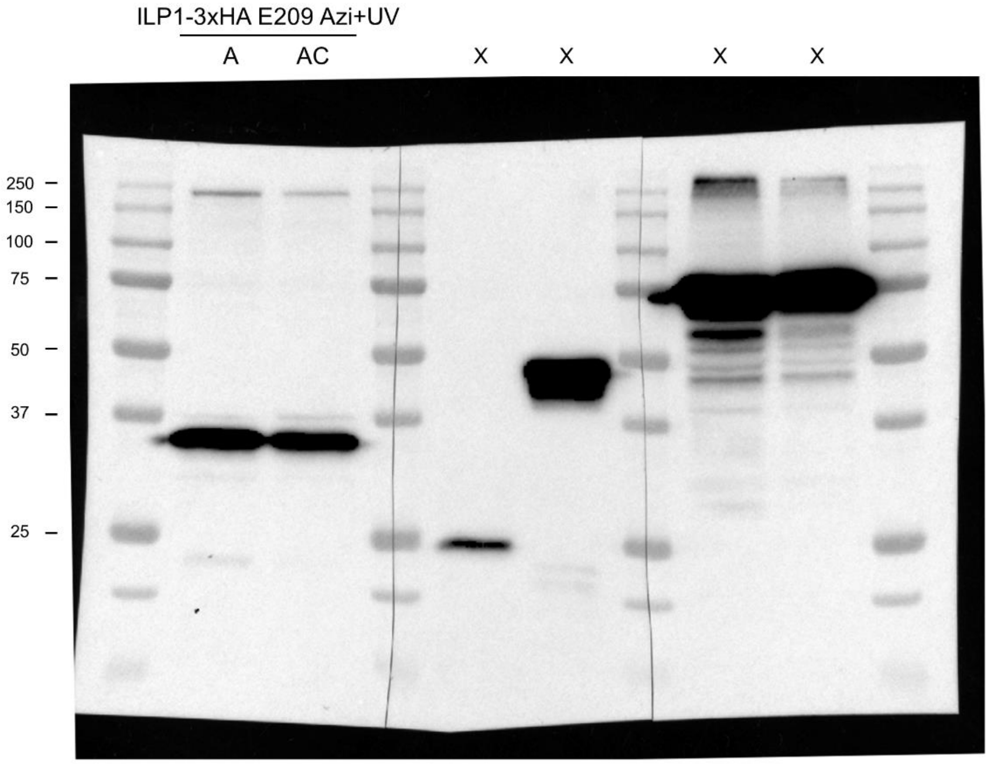

ILP1-HA blot

30 sec exposure anti-HA IP

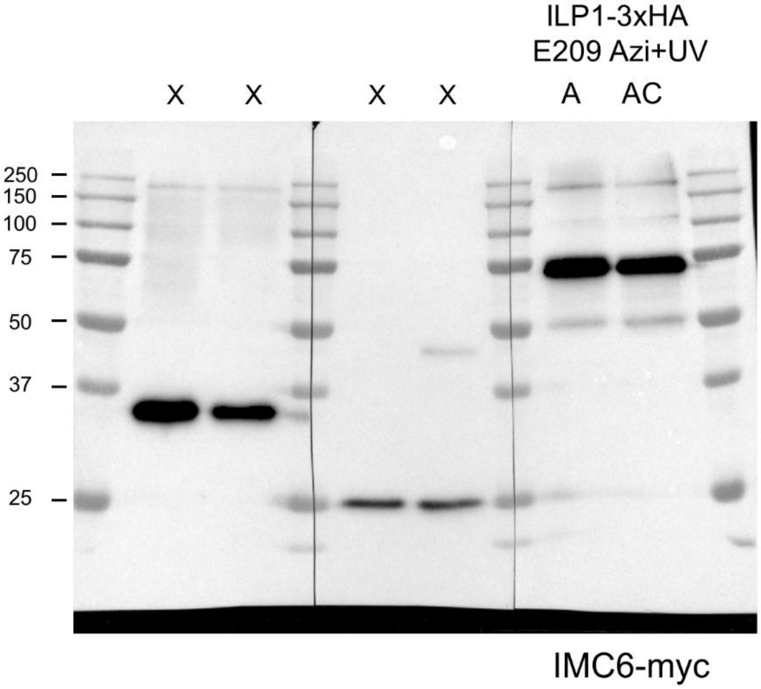

120 sec exposure anti-HA IP

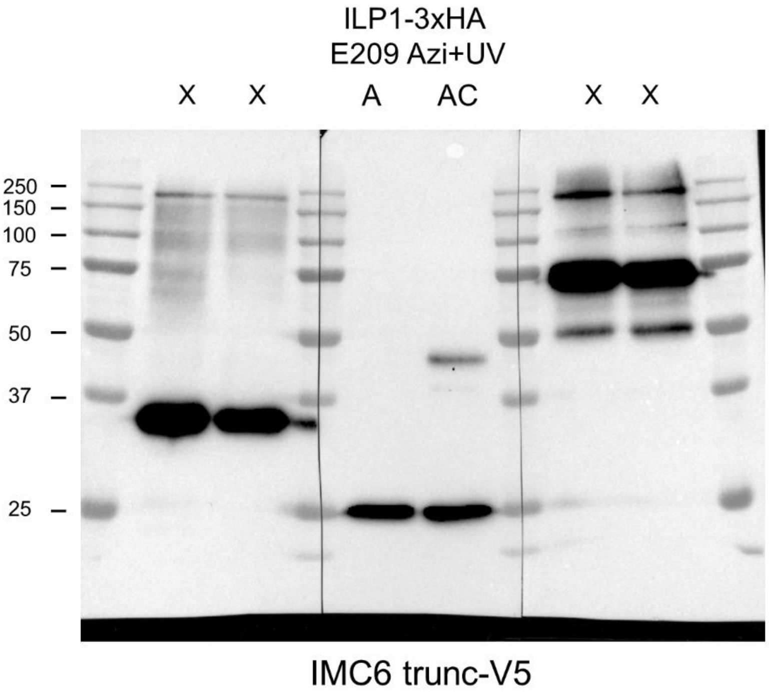

200 sec exposure anti-HA IP

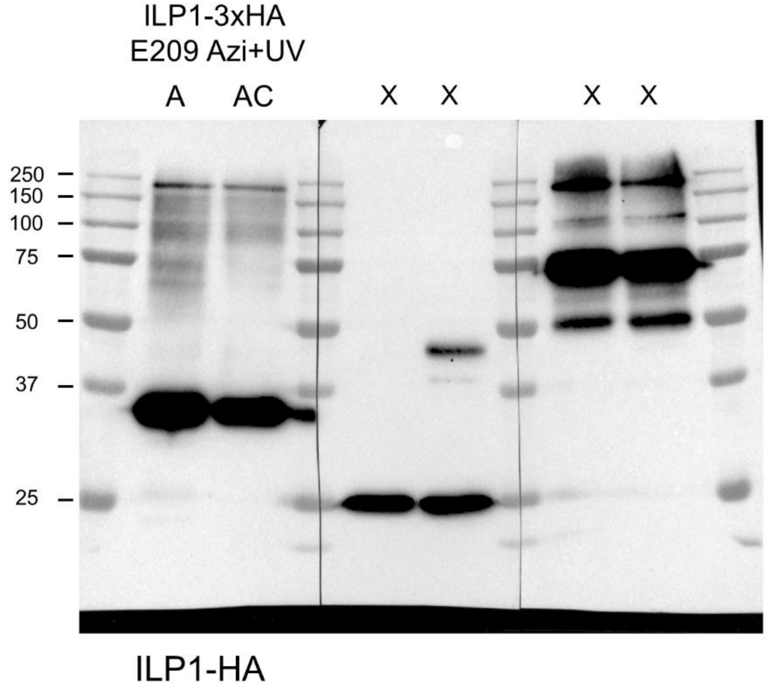

30 sec exposure anti-HA IP

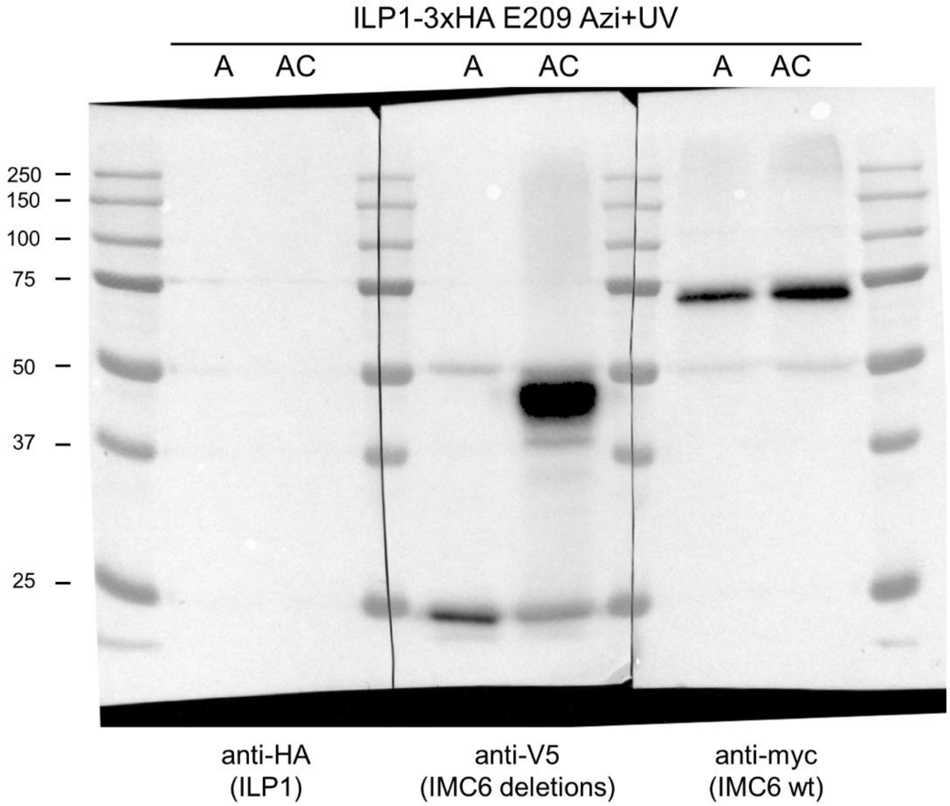

N+A strain whole cell lysate

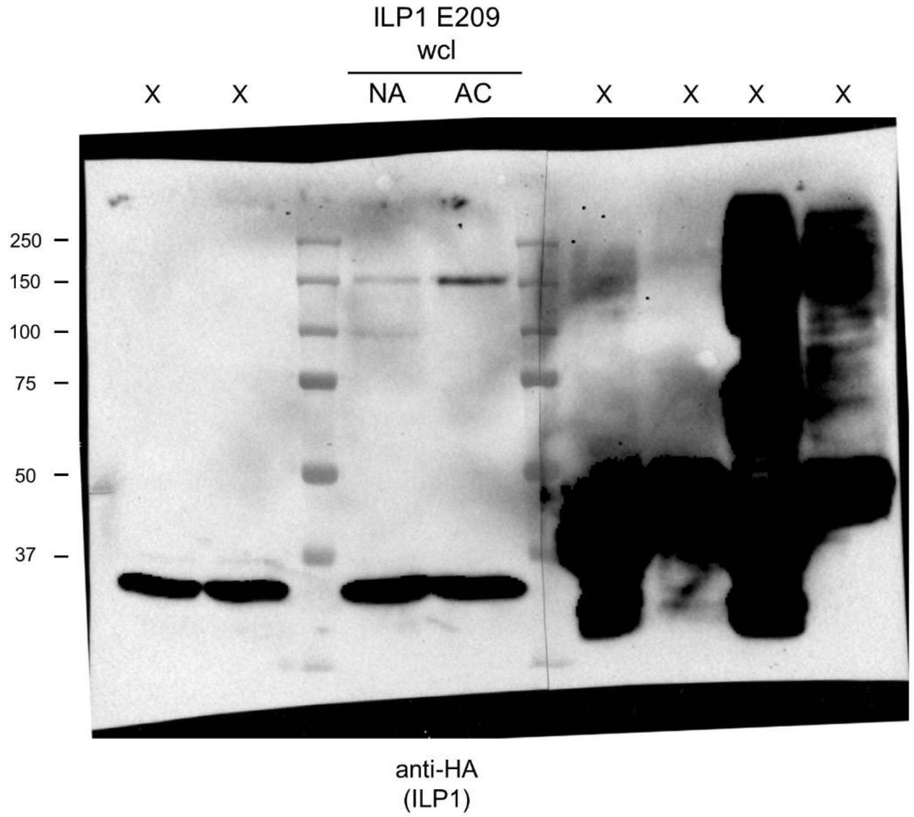

N+A strain anti-HA IP

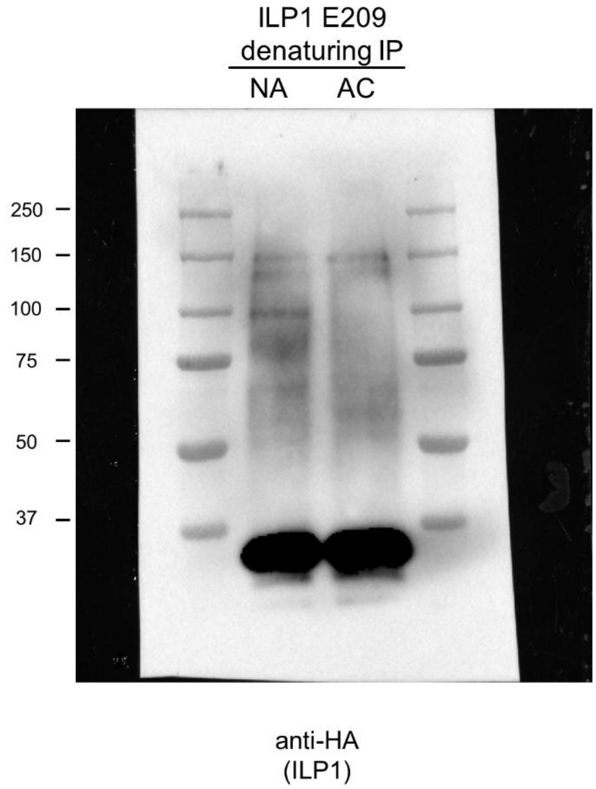

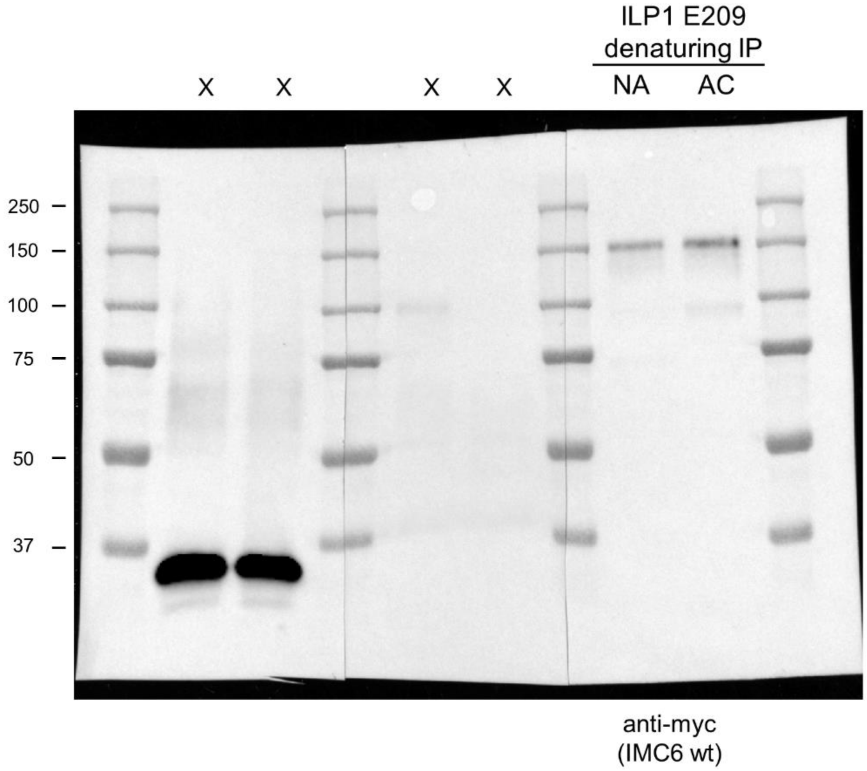

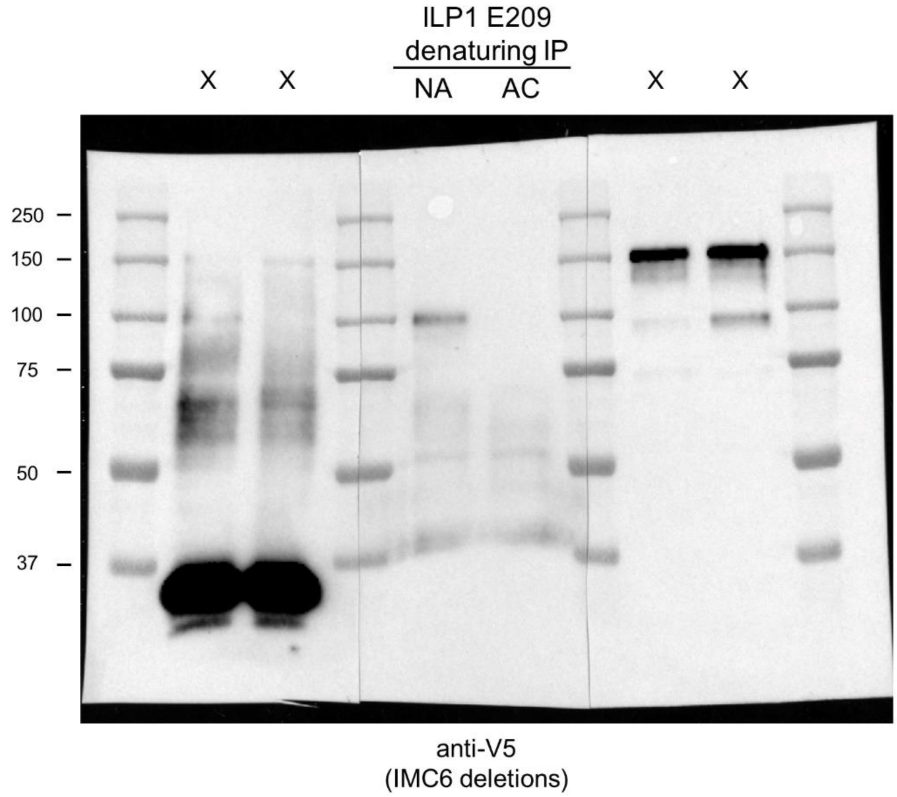
ILP1 E209
denaturing IP
X
X
NA
AC
X
X
250
150
100
75
50
37
anti-V5
(IMC6 deletions)

Figure 8

ILP11 Y160 large scale crosslinking IP

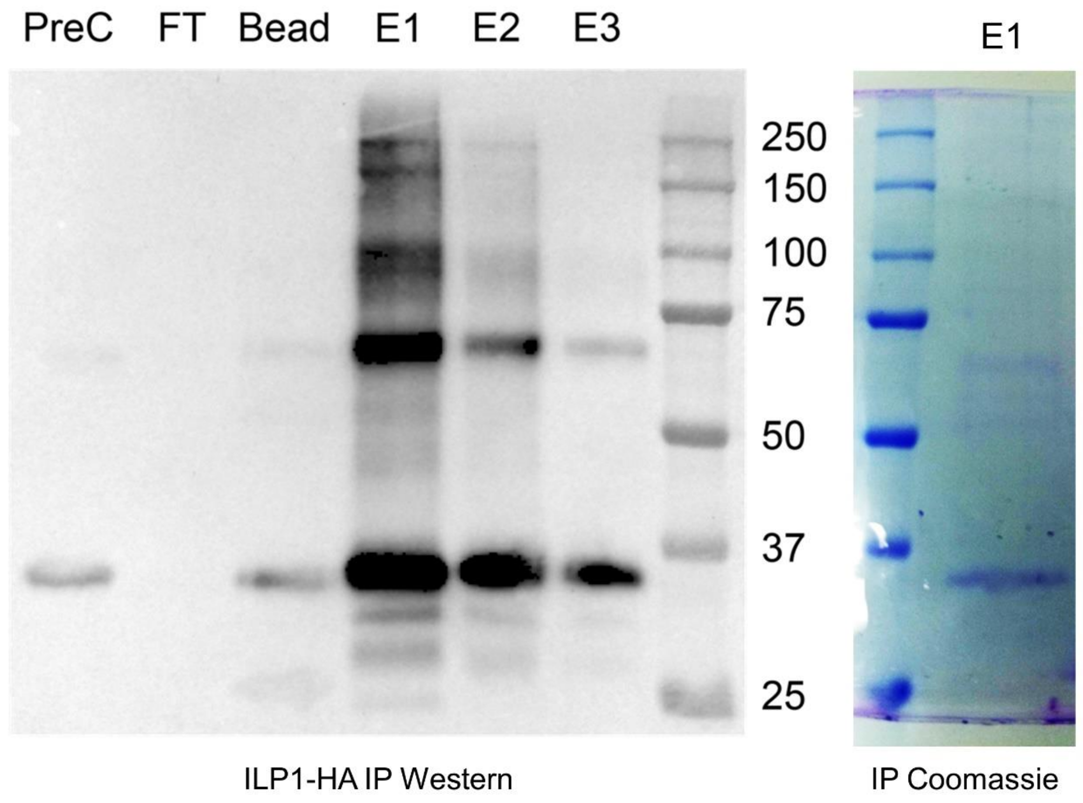

IMC27 fractionation anti-HA blot

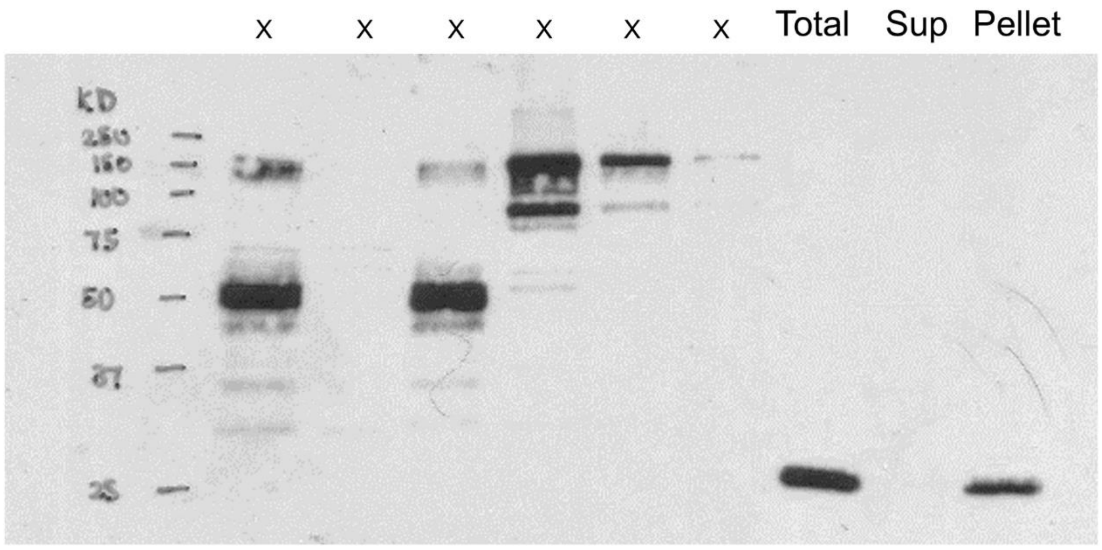

IMC27 fractionation anti-IMC1 blot

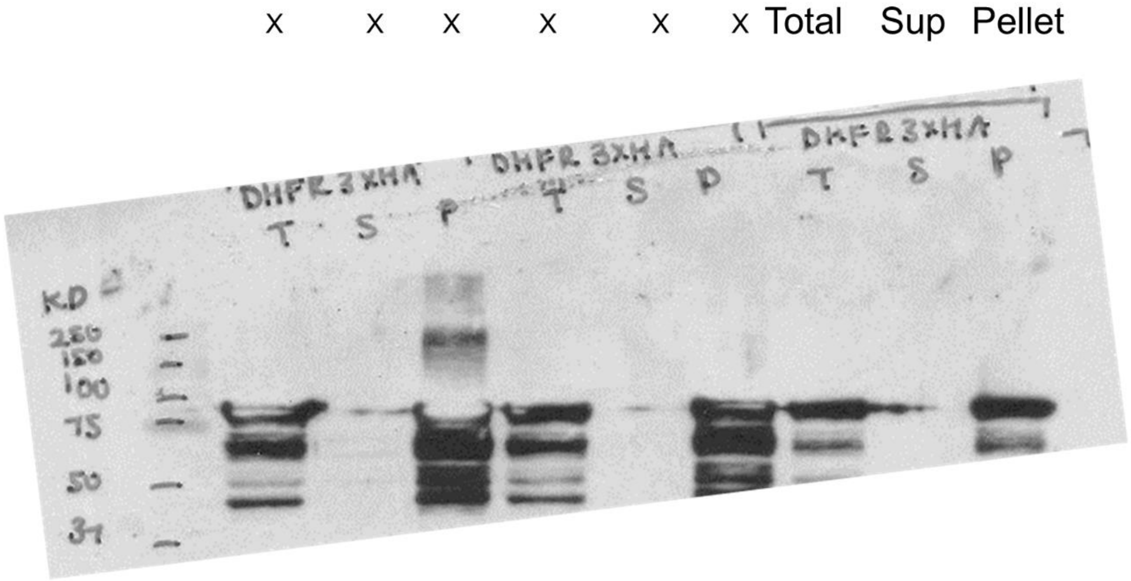

IMC27 fractionation anti-ISP3 blot

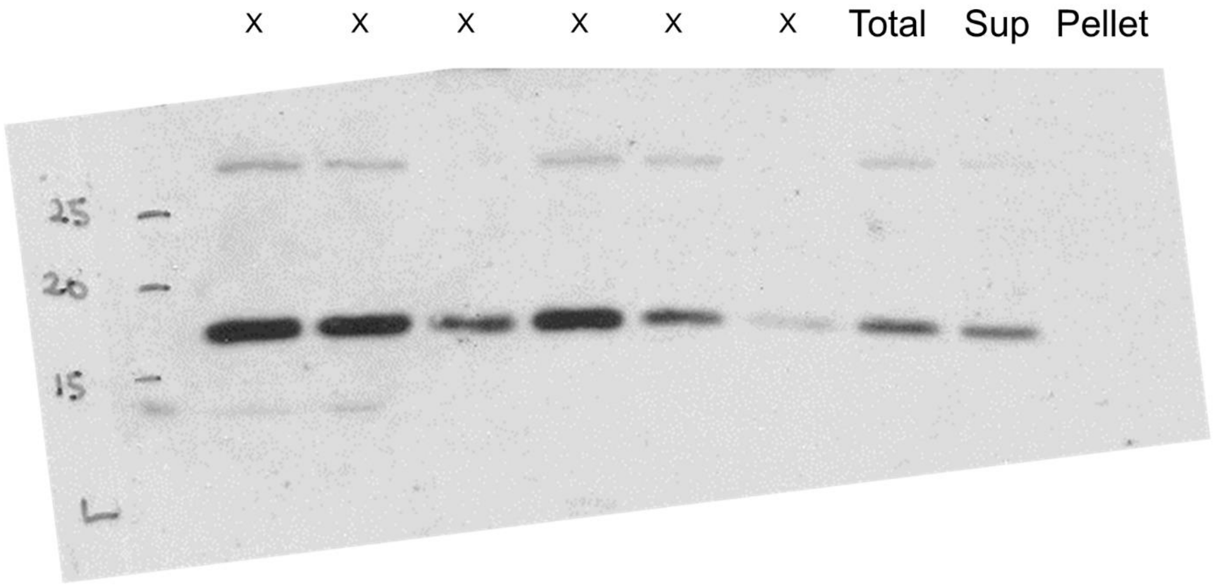

ILP1-IMC27 whole cell lysate

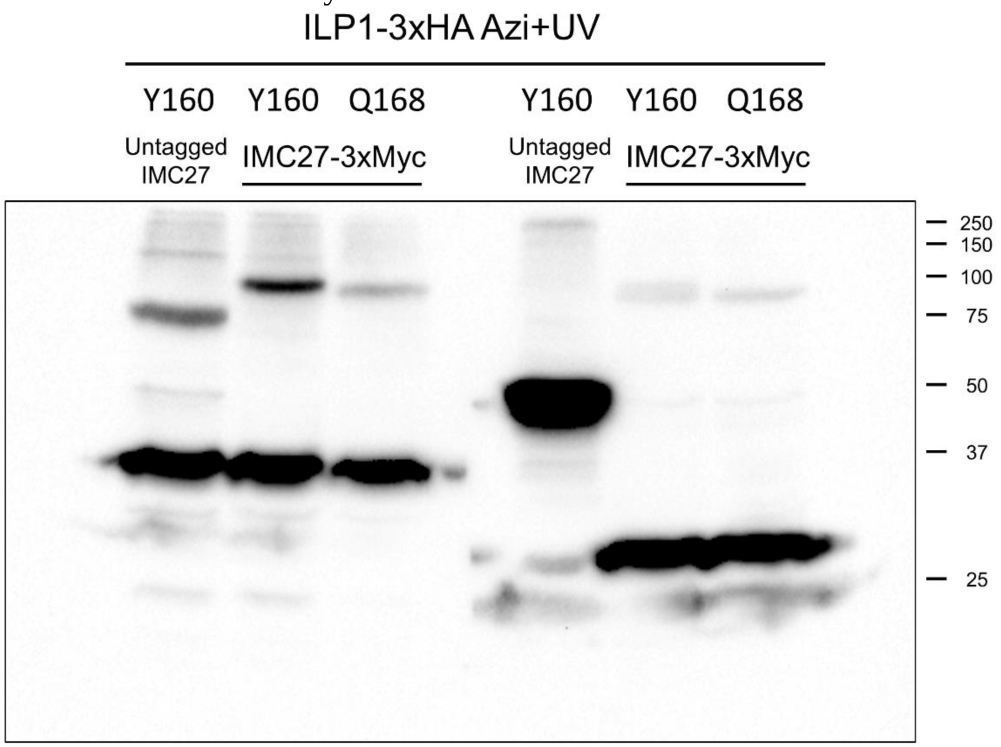

ILP1-IMC27 denaturing IP

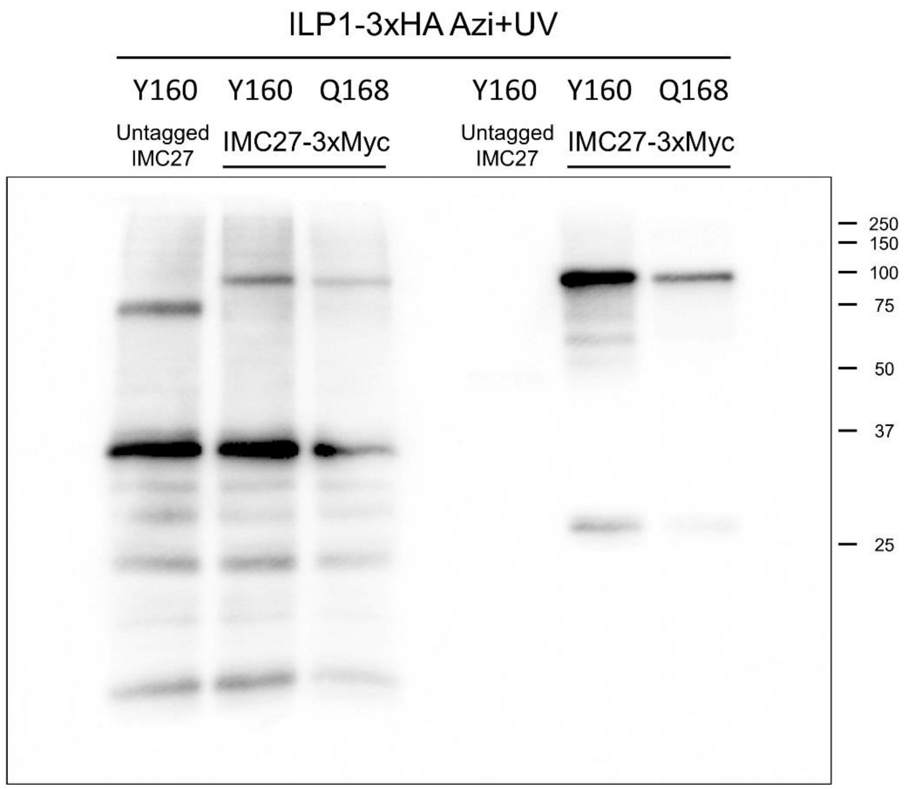

S2 Fig

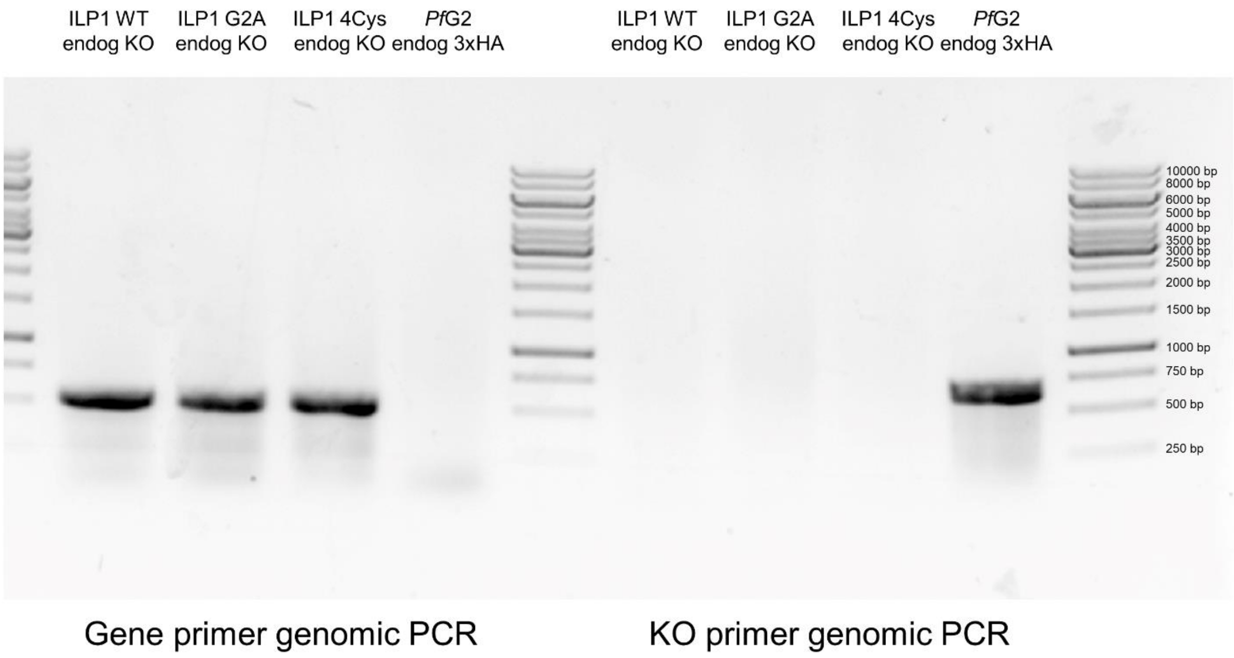
ILP1 WT endog KO
ILP1 G2A endog KO
ILP1 4Cys endog KO
*Pf*G2 endog 3xHA
ILP1 WT endog KO
ILP1 G2A endog KO
ILP1 4Cys endog KO
*Pf*G2 endog 3xHA
10000 bp
8000 bp
6000 bp
5000 bp
4000 bp
3500 bp
3000 bp
2500 bp
2000 bp
1500 bp
1000 bp
750 bp
500 bp
250 bp
Gene primer genomic PCR
KO primer genomic PCR

S3 Fig

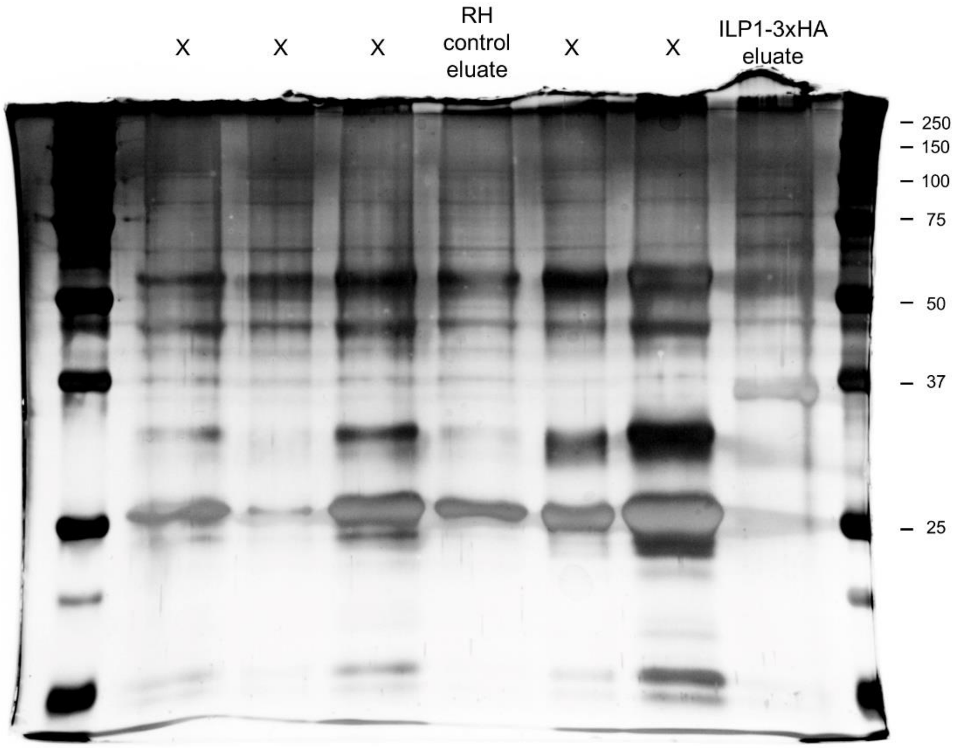
X
X
X
RH control eluate
X
X
ILP1-3xHA eluate
250
150
100
75
50
37
25
